# Comparative Geometrical Analysis of Leucine-Rich Repeat Structures in the Nod-Like and Toll-Like Receptors in Vertebrate Innate Immunity

**DOI:** 10.3390/biom5031955

**Published:** 2015-08-18

**Authors:** Norio Matsushima, Hiroki Miyashita, Purevjav Enkhbayar, Robert H. Kretsinger

**Affiliations:** 1 The Institute of Tandem Repeats, Sapporo 060-8556, Japan; E-Mail: miya_hokkaido@hotmail.com; 2 Department of Information and Computer Science, School of Engineering and Applied Sciences, National University of Mongolia, Ulaanbaatar 210646/377, Mongolia; E-Mail: enkhb2003@yahoo.com; 3 Department of Biology, University of Virginia, Charlottesville, VA 22904, USA; E-Mail: rhk5i@virginia.edu

**Keywords:** TLRs, NLRs, receptor, RP105, CD14, helix, dimer

## Abstract

The NOD-like receptors (NLRs) and Toll-like receptors (TLRs) are pattern recognition receptors that are involved in the innate, pathogen pattern recognition system. The TLR and NLR receptors contain leucine-rich repeats (LRRs) that are responsible for ligand interactions. In LRRs short β-strands stack parallel and then the LRRs form a super helical arrangement of repeating structural units (called a coil of solenoids). The structures of the LRR domains of NLRC4, NLRP1, and NLRX1 in NLRs and of TLR1-5, TLR6, TLR8, TLR9 in TLRs have been determined. Here we report nine geometrical parameters that characterize the LRR domains; these include four helical parameters from HELFIT analysis. These nine parameters characterize well the LRR structures in NLRs and TLRs; the LRRs of NLR adopts a right-handed helix. In contrast, the TLR LRRs adopt either a left-handed helix or are nearly flat; RP105 and CD14 also adopt a left-handed helix. This geometrical analysis subdivides TLRs into four groups consisting of TLR3/TLR8/TLR9, TLR1/TLR2/TRR6, TLR4, and TLR5; these correspond to the phylogenetic tree based on amino acid sequences. In the TLRs an ascending lateral surface that consists of loops connecting the β-strand at the C-terminal side is involved in protein, protein/ligand interactions, but not the descending lateral surface on the opposite side.

## 1. Introduction

Nucleotide binding oligomerization domain (NOD-like) receptors (NLRs) and Toll-like receptors (TLRs) are pattern recognition receptors that together with RIG-I-like receptor (retinoic acid inducible gene 1) and C-type lectin families make up the innate, pathogen pattern recognition system [1,2,3,4,5,6,7,8].

**Figure 1:**
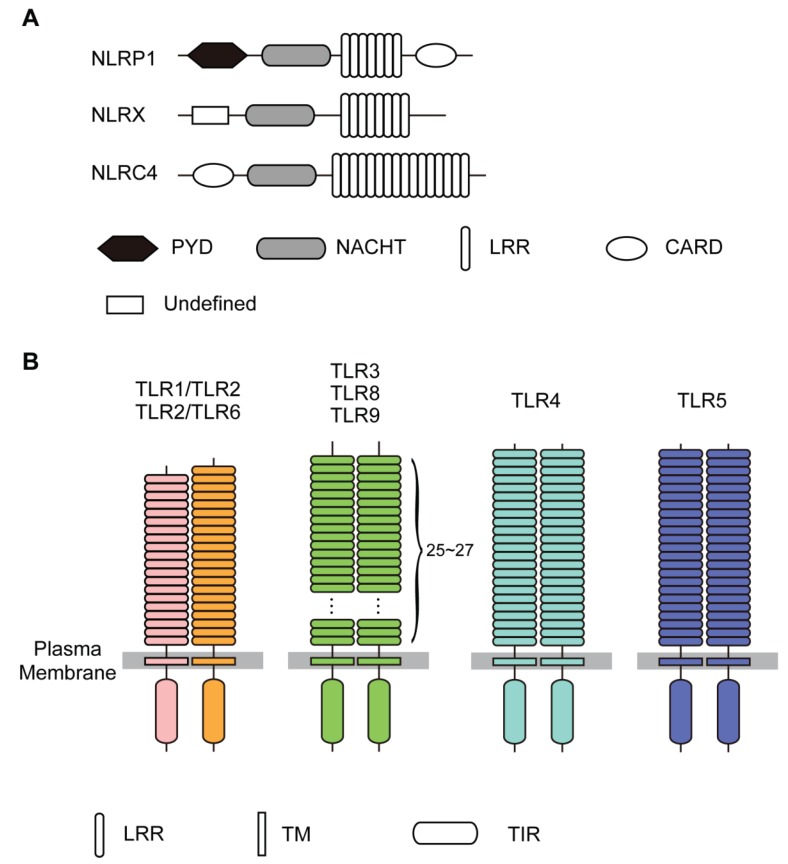
Domain architecture of NLRs and TLRs. (**A**) NLRP1, NLRX, and NLRC4; (**B**) TLR1/TLR2, TLR2/TLR6, TLR3 TLR8, TLR9, TLR4, and TLR5.

NLRs are soluble cytoplasmic proteins that mediate host innate immunity as intracellular surveillance sensors against common molecular patterns of invading pathogens [1,2,3]. The NLRs are subdivided into five subfamilies: **NLRP** (NLRP1-14), **NLRX** (NLRX1), **NLRC** (NOD1, NOD2, NLRC3, NLRC4, and NLRC5), **NLRA** (CIITA), and **NLRB** (NAIP). Some NLRs (such as NLRP1 and NLRC4) form a large multi-protein complex called an inflammasome, which cleaves procaspase-1 with the subsequent formation of activated caspase I. These proteins typically consist of a NACHT and other subdomains, an N-terminal effector-binding domain most commonly a CARD (caspase activation and recruitment domain), a BIR (baculovirus inhibitor repeat), or a PYD (pyrin) domain, and a C-terminal putative ligand-binding domain consisting of tandem LRRs (Figure 1).

Toll-like receptors (TLRs) are homologous to the *Toll* receptor, first discovered in the fruit fly *Drosophila* [9]. The Toll receptor plays a role in the development of the dorso-ventral body axis in *Drosophila*, acting as a cell surface receptor for the cytokine ligand, Spätzle [10]. Toll and related receptors also function in the innate immune response to fungal and bacterial infection in the fruit fly [11].

In vertebrates, TLRs also play a central role in innate immune defense against infection by binding to molecules from microbes [5,6,7,8]. The TLR family consists of 10 and 12 members in human and mice, respectively; various species of fish have at least 20 TLR members [12]. They are involved in the recognition of multiple groups of microbial molecules that are not usually found in humans and mice. TLRs 3, 7, 8, and 9 are localized in intracellular organelles and recognize various types of microbial nucleic acids. TLR2 forms heterodimeric complexes with either TLR1 or TLR6 that can bind to lipo-peptides or lipo-proteins from bacterial membranes. TLR4 forms hetero-dimers with MD-2 that are activated by lipo-polysaccharide (LPS) from gram-negative bacteria. TLR5 can be stimulated by the bacterial protein flagellin. A small molecule agonist of TLRs 7 and 8 is being used as an antiviral and anticancer drug [13].

TLRs are type I transmembrane (TM) proteins composed of extra-cellular leucine-rich repeats (LRRs) that mediate recognition of Pathogen-Associated Molecular Patterns (PAMPs) transmembrane domains, and cytoplasmic Toll interleukin (IL)-1 receptor (TIR) domains that interact with downstream adaptor proteins required for signaling (Figure 1); type I proteins have a single TM stretch of hydrophobic residues, with the portion of the polypeptide on the NH2-terminal side of the TM domain exposed on the exterior side of the membrane and the COOH-terminal portion exposed on the cytoplasmic side.

LRRs are present in over 98,000 proteins [14,15,16,17,18]. Most LRR proteins are involved in protein, protein interactions, as observed in the plant immune response and the mammalian innate immune response. Each repeat of LRRs is typically 20–30 residues long and can be divided into an HCS (Highly Conserved Segment) and VS (Variable Segment). The HCS part consists of LxxLxLxxNx(x/-)L, in which “L” is Leu, Ile, Val, or Phe; “N” is Asn, Thr, Ser, or Cys; “x” is a non-conserved residue; and “-” is a possible deletion site [14,15,19]. There are eight classes of LRRs—“RI-like”, “Typical”, “SDS22-like”, ”IRREKO”, “Bacterial”, “Plant specific”, “TpLRR”, and “Cysteine-containing” [15,20,21,22].

Tandem LRR domains consist of a super helical arrangement of repeating structural units and fold into a horse shoe, a right handed or left handed helix, or a prism shape [23]. Three residues at positions 3 to 5, xLx, in the HCS part form a short β-strand. These β-strands stack parallel and then the LRRs assume their super helical arrangements. The LRRs show the pattern of H-bonding (N-H → O=C) in the stack of parallel β-strands.

**Figure 2:**
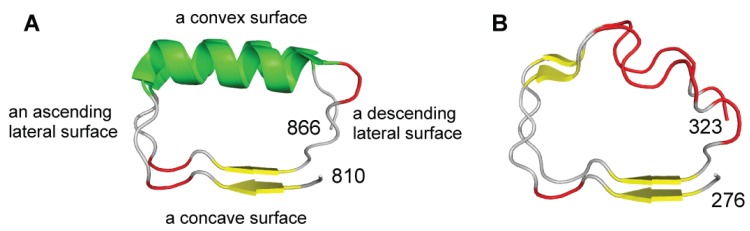
Secondary structures on the convex sides of LRRs in representatives of NLRs and TLRs. (**A**) Ribbon diagram of two consecutive LRRs (LRR1 and LRR2) in human NLRP1, 1473 residues long (*n* = 7), sequence 810-LKELDLSGNSLSHSAVKSLCKTLRRPRCLLETLRLAGCGLTAEDCKDLAFGLRANQT-866 [4IM6]; (**B**) Ribbon diagram of two LRRs (LRR11 and LRR12) in human TLR3, 904 residues long, (*n* = 25), sequence 276-LTMLDLSYNNLNVVGNDSFAWLPQLEYFFLEYNNIQHLFSHSLHGLFN-323 [1ZIW_A]. In all figures, two consecutive LRRs are shown. Yellow arrows represent β-strands or extended conformation, green ribbons α-helices, and red tubes β-turns. Throughout the text, loops connecting the concave side to the convex side are referred to as “ascending”, and the ones connecting the convex side to the concave side are referred to as “descending”.

The concave face, consisting of the HCSs of each LRR unit, forms a parallel β-sheet, with one strand from each HCS unit [16,17]. The convex face, consisting of the VS of each LRR unit, is made of a variety of secondary structures including the α-helix, 3_10_-helix, poly-proline II helix, an extended conformation or a tandem arrangement of β-turns. The various secondary structures on the convex side are connected to the strands forming the β-sheet on its concave side by two loops. The “ascending loop” links the C-terminal end of the β-strand in the HCS to the N-terminus of the characteristic secondary structure in the VS (Figure 2) [18]. The “descending loop” links the C-terminal end of the characteristic secondary structure in the VS to the N-terminus of the β-strand in the HCS of the following unit. Finally, each LRR domain contains a concave surface, a convex surface, an ascending lateral surface, and a descending lateral surface on the opposite side. Most of the known LRR structures have an N-cap and/or C-cap that shields the hydrophobic core of the first LRR unit at the N-terminus and/or the last unit at the C-terminus.

The program HELFIT determines the helical parameters, such as helix axis, pitch (with handedness), radius, and number of points or units per turn, as well as rmsd [24,25]. We calculated helical parameters of 642 LRRs of known structure from 114 proteins by HELFIT analysis [23]. The helical parameters of the eight LRR classes frequently overlap one another. However, the constant distance between parallel β-strands is the primary determinant of the helical parameters of the LRRs. The helical parameters of the LRRs unambiguously describe both right-handed and left-handed helices, helical dimers, and subdomains, if they exist. Most LRRs form right-handed helices.

There have been many reviews of the LRR structures of TLRs and NLRs [6,7,8,26,27,28,29,30,31,32]. However, there is no geometric analysis to provide quantitative comparison of the 3D structures. After our recent paper on the HELFIT analysis of the known LRR structures [23], many PDB files of other LRR domains were added to RCSB Protein Data Bank and Protein Data Bank Japan (PDBj). Structural data of TLRs and NLRs have also increased.

Here we focus on NLRs and TLRs and calculate nine geometrical parameters of all of their known LRR structures together with secondary structure analysis of individual LRR units. These nine parameters well characterize the LRR structures in NLRs and TLRs. The *Drosophila* Toll receptor as well as NLRC4, NLRP1, and NLRX adopt a right handed helix, while TLR1-5, TLR6, TLR8, and TLR9 adopt either a left handed helix or are nearly flat. This geometrical analysis subdivides TLRs into four groups consisting of TLR3/TLR8/TLR9, TLR1/TLR2/TRR6, TLR4, and TLR5. In the TLRs an ascending lateral surface, but not the descending lateral surface, is involved in protein, protein/ligand interactions.

## 2. Analysis Methods

### 2.1. Structural Data

We collected 56 pdb files of NLRC4, NLRP1, NLRX1, TLR1-5, TLR6, TLR8, TLR9, RP105, and CD14 for the present analysis. We analyzed 115 different chains (Supplementary Table S1) [23].

### 2.2. Structural Secondary Structure Analysis of LRRs

First, secondary structure assignment were made from the atomic coordinates of the LRR structures by DSSP [33]. Second, individual repeat units in the LRR domains were identified by analysis of the secondary and tertiary structures. Finally, sequence alignments of individual LRRs were made by eye.

### 2.3. Geometrical Analysis

**Figure 3:**
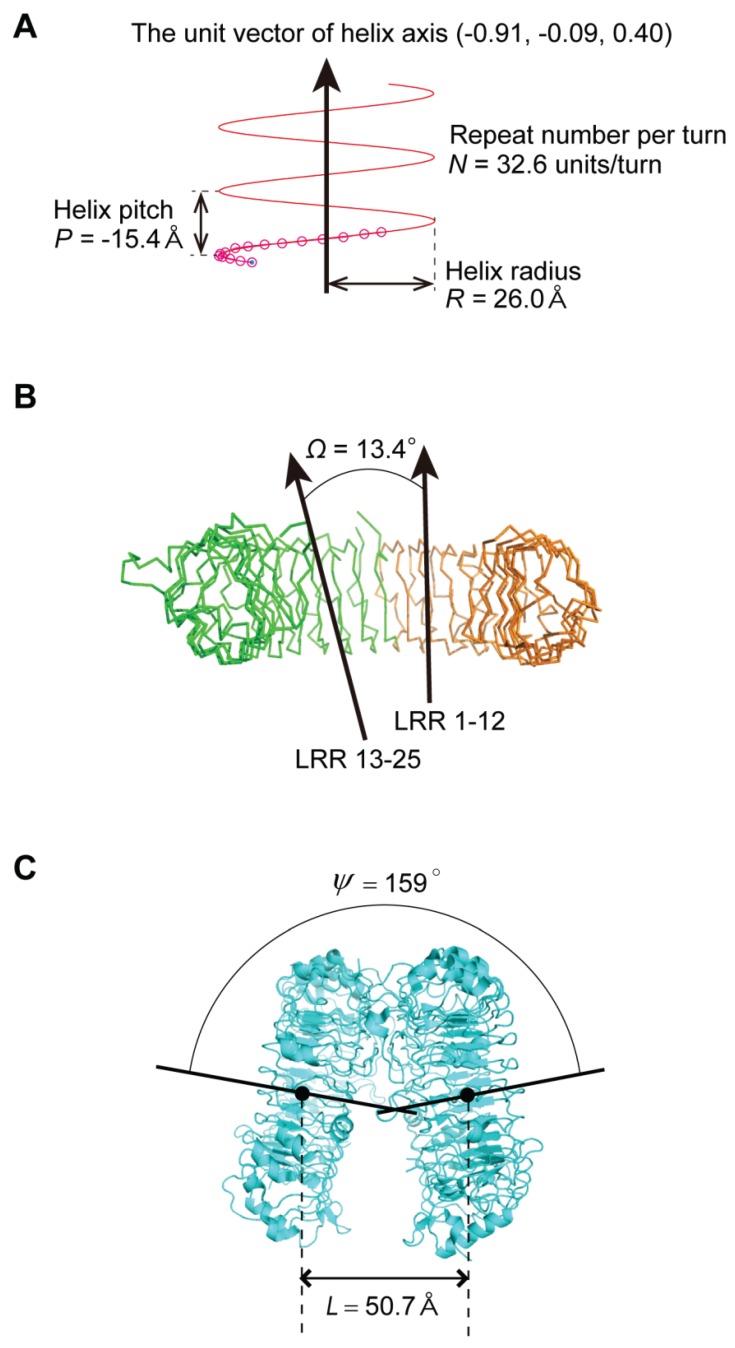
Nine parameters characterizing LRR domains. (**A**) Four helix parameters (the unit vector of helix axis, helix pitch, helix radius, repeat number per turn) calculated by HELFIT in helical LRRs in zebrafish TLR5 with *n* = 16 [3V47_A]; (**B**) The angle between the axes of the two helices of the two subdomains in LRRs with two subdomains (Ω) in human TLR3 with *n* = 25 [1ZIW_A]; (**C**) The distance between the two helix axes of the two monomer (*L)* in dimers and the angle between the two helix axes (ψ) in human TLR8 homo-dimers in the free state with *n* = 27 [3W3G]. The direction of the helix axis is in the direction of the arrows.

A helix consisting of *n* repeat units may be characterized by helix axis, pitch (*P*) (with handedness), helix radius (*R*), and number of repeat units per turn (*N*). HELFIT computes these five parameters in which the helix axis is represented by the unit vector (Figure 3); it requires two parameters to describe the helix axis after the helix has been translated to the origin of the coordinate system [24,25]. These parameters also yield the rise per repeat unit (Δz = *P*/*N*) and the rotation per repeat unit (ΔΦ = 360°/*N)*. Moreover, HELFIT gives *rmsd*:

$$rmsd={\left[\;\text{the}\;\text{minimum}\;\text{of}\;\frac{\sum {\left(d_{i}\right)}^{2}}{n}\;\right]}^{\frac{1}{2}}$$

where *d_i_* is the closest distances from data point to the trace of the helix. Here *p* (= *rmsd*/(*n* − 1)^½^) gives the regularity of the helix independent of its length. In most LRRs the three residues at positions 3–5 in the HCS form a short β-strand, which is almost completely conserved in all LRR structures. These β-strands from adjacent repeat units with the pattern of H-bonding (N-H → O=C) form a parallel, pleated sheet on the inside, or concave surface of the helix. As reference points for HELFIT we use the coordinates of the α-carbon (Cα) of the consensus leucine residue at position 4 (corresponding to the middle of each β-strand) in individual LRR repeat units.

If *p* is relatively large (>0.2 Å), the LRR is not well described by a helix and might be more easily visualized as another shape, e.g., an ellipse, or might consists of multiple subdomains [23]. There are some LRR domains consisting of two or three subdomains. The LRR domains may be characterized by the angle, Ω, between the two helix axes of the two subdomains (Figure 3) [23]. Moreover, LRR domains sometimes form dimers or tetramers. The spatial arrangement may be characterized by the distance and the angle between the two helix axes of the two monomers in the dimers, *L* and ψ (Figure 3) [23]. The 3D circle fitting was also utilized for calculating the average distance between adjacent repeats by the helical parameters. “L” was calculated from the center of the circle determined by the 3D circle fitting. Finally, the nine parameters—*P*, Δz, ΔΦ, *N*, *R*, *p*, Ω, *L*, and ψ—were calculated.

## 3. Structures of NLRs

### 3.1. The LRRs in NLRs

The crystal structures of NLRP1, NLRX, and NLRC4 have been determined [34,35,36]. The LRRs of NLRP1, NLRX1, and NLRC4 belong to the “RI-like” class [23]. The consensus unit is LxxLxLxx(N/C)xLxxxgoxxLxxoLxxzxxx with typically 28 or 29 residues. Consensus, hydrophobic residues are responsible for the proper packing of the hydrophobic core.

Human NLRP1 with 1473 residues contains seven LRRs units; the last repeat has only the HCS. Six of the seven LRRs consist of alternating 29 and 28/27 residues each and thus they form higher order repeating units; this feature is also observed the LRRs in ribonuclease inhibitor (RI), mice MATER and human RNO2 [16]. The NLRP1 LRRs belong to the “RI-like” class. Three residues at positions 3 to 5, xLx, in the HCS part form a short β-strand. All of the VSs adopt an α-helical conformation of 10–13 residues (β-α structural units) (Figure 2 and Supplementary Figure S1). Human NLRX1 with 975 residues contains eight LRRs. The eight unit lengths are 29, 25, 29, 28, 28, 28, 28 and 28. The structural unit is β-α.

Mouse NLRC4 is 1024 residues long and contains sixteen LRRs. The unit lengths range from 21 to 50. These LRRs are more variable than those in NLRP1 and NLRX1. However, most of the VSs adopt an α-helical conformation; only the sixth VS consists of four β-turns (Supplementary Figure S1).

### 3.2. Human NLRP1

**Figure 4:**
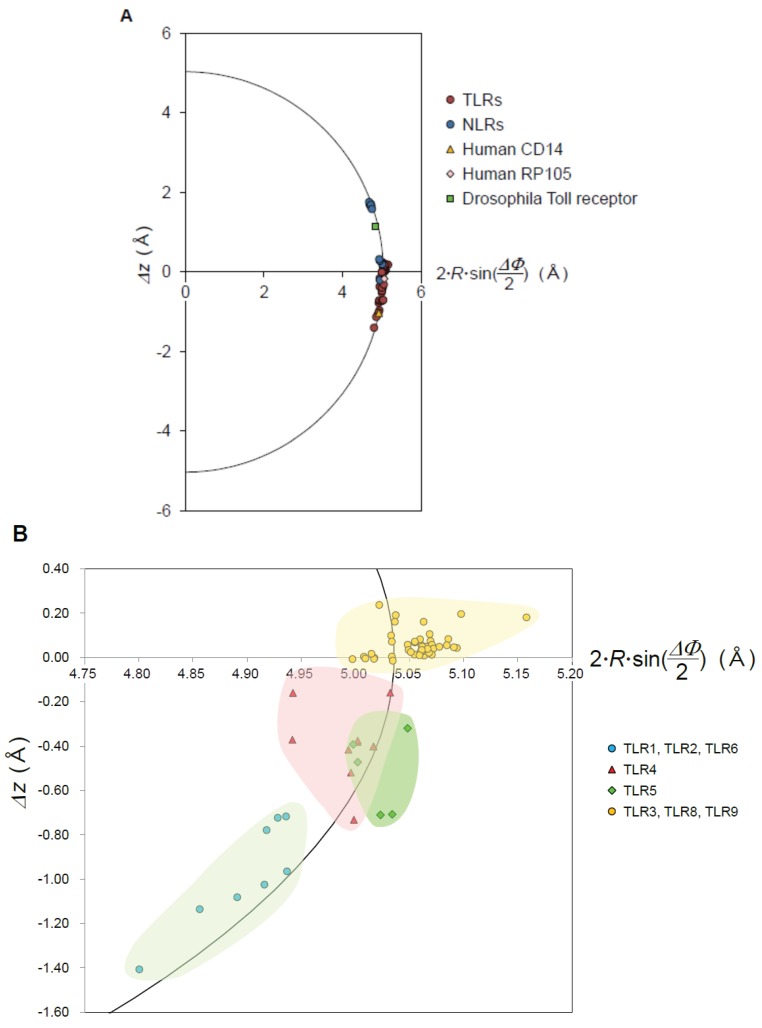
The correlation of Δz and 2·R·sin (ΔΦ/2) in the helix parameters. (**A**) Circular plot of the LRRs in NLRs (NLRP1, NLRX, and NLRC), TLRs (TLR3, TLR8, TLR9, TLR1, TLR2, TLR6, TLR4, and TLR5), RP105, CD14, and Drosophila Toll receptor; (**B**) Circular plot of the LRRs in the TLRs. “*R*” is the helix radius of a helix consisting of *n* repeat units, “Δz” the rise per repeat unit, and “ΔΦ” the rotation per repeat unit.

**Figure 5:**
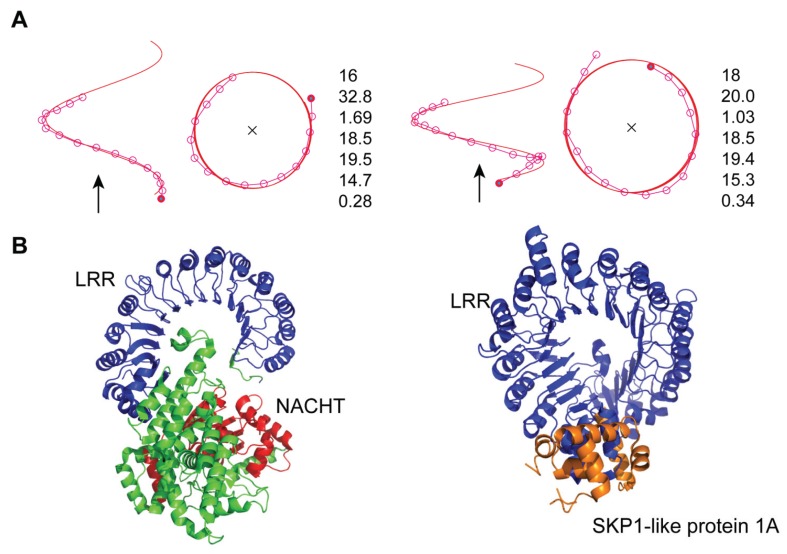
Three-dimensional structures of human NLRC4 [4KXF_A] and AtTIR1 [2P1M], (**A**) HELFIT analyses of the LRR domains; (**B**) Ribbon diagram. In all panels, left side shows human NLRC4 and right side shows AtTIR1. In panel A, seven parameters for each protein are in order from top to bottom: the repeat number n, the helix pitch *P* [Å], the rise per repeat unit Δ*z* [Å], the rotation per repeat unit ΔΦ [°], the number of repeat units per turn *N* [units/repeat], the helix radius *R* [Å], and the *p* parameter [Å]. Left and right figures of each protein indicate side views and views along the axes of a helix, respectively. In the side views the direction of helix axis is in the direction of the arrows. In the views along helix axes, the cross signs show that it is in a downward direction.

NLRP1 senses the presence of the bacterial cell wall component, l-muramyl dipeptide (MDP) inside the cell [37]. NLRP1 consists of five domains of PYD, NBD/NACHT, LRRs (*n* = 7), FIND, and CARD (Figure 1). The *p* value is very small: *p* = 0.03 Å (Supplementary Table S1). Thus the helix is regular. The helix parameters are *P* = 4.84 Å, Δz = 0.19 Å, ΔΦ = 14.33°, *N* = 25.13 units/repeat, and *R* = 20.09 Å (Figure 4). The LRR domain is nearly flat, as seen in RI (Figure 5). The *R* value is 2.0 Å larger than those of the RI LRR domains with *n* = 17.

### 3.3. Human NLRX1

NLRX1 is a mitochondrial protein and is a pro-inflammatory activator that stimulates the production of reactive oxygen species (ROS) via TNF-α activation [35]. Moreover, NLRX1 can also down regulate inflammatory responses as a negative regulator of RIG-1 and TLRs through interaction with the mitochondrial anti-viral signaling adaptor and TRAF6-IKK, respectively.

NLRX1 contains two domains of NBD/NACHT and LRRs (*n* = 8) (Figure 1). The structure of the LRR domain of hNLRX1 (residues 629–975) that directly interacts with RNA has been determined [35]. The LRR region from NLRX1 has an additional helical bundle C-terminal to the LRR (LRRCT). The LRR domain forms a trimer in the crystal. The three domains are related by a three-fold axis of rotation. The helix parameters range over: *P* = −6.31 → 10.06 Å, Δz = −0.21 → 0.32 Å, ΔΦ = 11.40 → 11.78, *N* = 30.56 → 31.58 units/repeat, and *R* = 24.10 → 24.84 Å (Figure 4 and Supplementary Table S1). The helix is regular; *p* = 0.08 → 0.09 Å. The LRR domain is nearly flat as seen in hNLRP1.

### 3.4. Mouse NLRC4

The NLRC4 inflammasome is activated in mice by bacterial flagellin or the components of type 3 secretion systems (T3SS) [36]. NLRC4 contains CARD, NBD/NACHT, and LRRs (*n* = 16) (Figure 1). The crystal structure of mouse NLRC4 (mNLRC4) mutant with the CARD (residues 1–89) and the internal residues (622–644) deleted (mNLRC4ΔCARD) [36]. The mNLRC4ΔCARD molecule forms a homo-octamer in the crystal. NLRC4 exists in an auto-inhibited/closed form with a bound ADP molecule residing in the center of the closed NOD domain. The helix parameters range over: *P* = 32.12 → 34.14 Å, Δz = 1.58 → 1.78 Å, ΔΦ = 17.73 → 18.51°, *N* = 19.45 → 20.38 units/repeat, and *R* = 14.67 → 15.40 Å (Figure 4 and Supplementary Table S1). The *p* values are large; *p* = 0.26 → 0.45 Å. The entire LRR adopts a right-handed helix with one full turn. However, the helix is distorted; the LRR domain (*n* = 16) form two subdomains, LRRs 1–8 and LRRs 9–16 and the average Ω is 16.3° (Supplementary Table S2).

It is interesting to compare the mMLRC4 structure with the structures of the *Arabidopsis* F-box proteins TIR1 (AtTIR1) and AtCOI1 (CORONATINE INSENSITIVE 1) which are homologues; TIR1 and COI1 are receptors for the plant hormones, auxin and jasmonate, respectively [38,39,40]. These two proteins form a SCF E3 ubiquitin ligase complex together with SKP1-like protein 1A (SKP1A). The structures of AtTIR1 and AtCOI1 in complex with SKP1A have been determined [38,39,40]. The LRRs (*n* = 18) belong to the “Cysteine-containing” class; the structural unit is β-α. Interestingly, the helical parameters and the large *p* value in mMLRC are comparable to those in AtTIR1 and AtCOI1 (Figure 5) [23,38,39,40]. The LRR domains form two subdomains, LRRs 1–8 and LRRs 9–18. Consequently, the entire LRR in AtTIR1 and in AtCOI1, as well as that in NLRC4, a distorted right handed helix with one full turn. In addition, the spatial arrangement of the NBD domain in mNLRC4ΔCARD is similar to that of SKP1A in AtTIR1/AtCOI1 (Figure 5). This may reflect convergent evolution.

## 4. Structures of TLRs

### 4.1. The LRRs in TLRs

The structures of TLR1-6, TLR8, and TLR9 have been determined [41,42,43,44,45,46,47,48,49,50,51,52,53,54,55,56,57,58,59]. The LRRs belong to the “Typical” class [60]. The consensus unit of the “Typical” class is LxxLxLxxNxLxxLpxxoFxxLxx with typically 24 residues. Hagfish Variable Lymphocyte Receptors B59 (VLRB) contains seven LRRs. Five of the seven repeats (LRR2-LRR6) are completely consistent with the consensus. All of the VS part forms a tandem arrangement of three β-turns that occurs frequently in sequences of the underlined residues of LxxLxLxxNxLxxLpxxoFxxLxx (Figure 2). The consecutive β-turns form a flat amphipathic structure with main chain hydrogen bonds in a linear arrangement. Thus, the structural unit is β-β_t_.

Among TLRs, TLR3 has the most LRRs that are very similar to the “Typical” consensus (Supplementary Figure S1). Correspondingly, the VS’s adopt a tandem arrangement of β-turns. TLR8 appears to have the second most “Typical” LRRs. In addition to the tandem β-turns, the VS’s frequently adopt a 3_10_-helix. In contrast, the LRRs in TLR1, TLR2, and TLR6 are highly variable. The occurrence of tandem β-turns in the VS’s is less frequent. Instead, α-helices and 3_10_-helices frequently occur. Moreover, the LRRs in TLR4 are relatively variable. The VS’s adopt tandem β-turns, 3_10_-helices, α-helices, and β-strands.

The short β-strand of three residues in the HCS part is highly conserved in TLR3, TLR8, and TLR9 (Figure 2). However, 8–9 LRRs in the central part of the domain adopt long β-strands consisting of 4 → 10 residues in TLR1, TLR2, TLR4, and TLR6 (Supplementary Figure S1). The descending loops frequently include one β-turn at the C-terminal side of the VS; the two underlined residues of LxxLxLxxNxLxxLpxxoFxxLxx (Figure 2). The ascending loops sometimes include one β-turn at the first two residues and one β-strand at the second two residues, which are at the underlined residues of LxxLxLxxNxLxxLpxxoFxxLxx. The latter β-strand occurs frequently as a pair in two consecutive repeats and forms H-bonding (N-H → O=C).

The sequence alignments of individual LRRs show that in TLRs most of the descending loops consist of five residues (Supplementary Figure S1). In contrast, the length of the ascending loop ranges to 8 to 24 residues. Thus, the ascending loops adopt a variety of conformations.

The data points of 2·*R*·sin (ΔΦ/2) *versus* Δ*z* fall on a circle with radius *D* (=5.04 Å) in all of the NLRs and TLRs, demonstrating the constant distance between parallel β-strands (Figure 4). *D* is the average Cα(*i*)–Cα(*i* + 1) distance between adjacent repeats, in which the Cα atoms are at position 4 in the β-strand. *D* is a function of Δz, ΔΦ, and *R*.

(1) $$D={\left[{\left\{2\cdot R\cdot \text{sin}(\frac{\Delta \Phi }{2})\right\}}^{2}+{\left(\Delta z\right)}^{2}\right]}^{\frac{1}{2}}$$

Figure 4B shows that the LRR domains of TLRs may be classified into four groups. The first group is TLR3, TLR8, and TLR9. The second is TLR1, TLR2, and TLR6. The third is TLR4; and the fourth is TLR5. These groups are closely related to the phylogenetic tree of TLRs based on their amino acid sequences [60,61,62,63].

The *p* values of the LRRs in the TLRs are all large; *p* > 2.0 Å (Supplementary Table S1). The helices are distorted. The large *p* values and the secondary structure analysis show that the LRRs in TLR3, TLR8, and TLR9 form two subdomains, while, those in TLR1, TLR2, TLR4, hTLR5, and TLR6 form three subdomains rather than two (Supplementary Table S2) [6].

The N-terminal LRR domains in TLR8 and TLR9 contain three tandem repeats of a super motif of ***STT*** in which “***S***” is a “Bacterial” LRR unit and “***T***” is a “Typical” LRR unit, as seen in the family of small leucine rich repeat proteoglycans (SLRPs) such as biglycan and decorin or the FLRT family [60,64,65]. The structures of biglycan, decorin, FLRT2, and FLRT3 have been determined [66,67,68]. These super LRR domains adopt right handed helices [23]. The helix parameters range over: *P* = 36.7 → 51.8 Å, Δz = 0.82 → 1.84 Å, ΔΦ = 9.6 → 14.3°, *N* = 25.3 → 37.4 units/repeat, and *R* = 19.0 → 29.0 Å.

### 4.2. TLR3, TLR8, and TLR9

#### 4.2.1. The Human and Mouse TLR3; Human TLR8; and Horse, Cow and Mouse TLR9

mTLR3 and hTLR3 (*n* = 25) has *P* = 2.22 → 7.61 Å and *N* = 32.14 → 33.06 units/repeat (Figure 4 and Supplementary Table S1). hTLR8 (*n* = 27) has *P* = 0.20 → 4.81 Å and *N* = 29.77 → 30.12 units/repeat. TLR9 from horse, cow and mouse (*n* = 27) has *P* = −0.45 → 2.16 Å and *N* = 30.29 → 30.70 units/repeat. The overall shape of the entire LRR is well approximated as three quarters or a full turn of an ellipse. All of the *P*’s are small and thus the LRR arcs are nearly flat. The LRR arc in TLR7 may be also nearly flat; although the structure of the ecto-domain is not known.

The LRRs in TLR8 and TLR9 form two subdomains; Ω = 11.12 → 23.04° (Supplementary Table S2). These small Ω values reflect the flat overall shape. The first subdomain, LRRs 1–15, adopts a right-handed helix; while, the second subdomain, LRRs 16–27 is near flat (Supplementary Table S1). The two subdomains in TLR3 show an inverse relation in the helical parameters.

#### 4.2.2. The TLR3—dsRNA Complex

**Figure 6:**
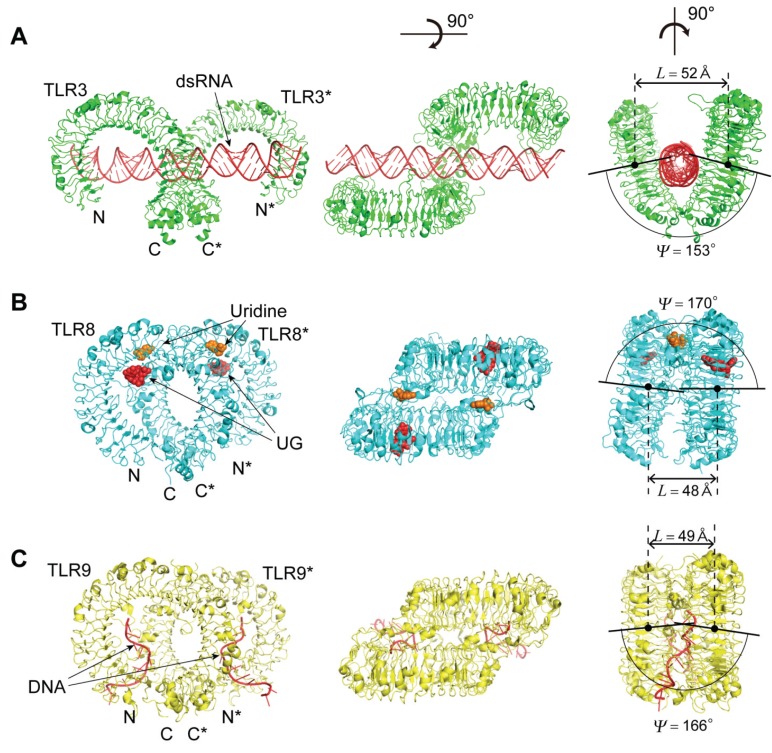

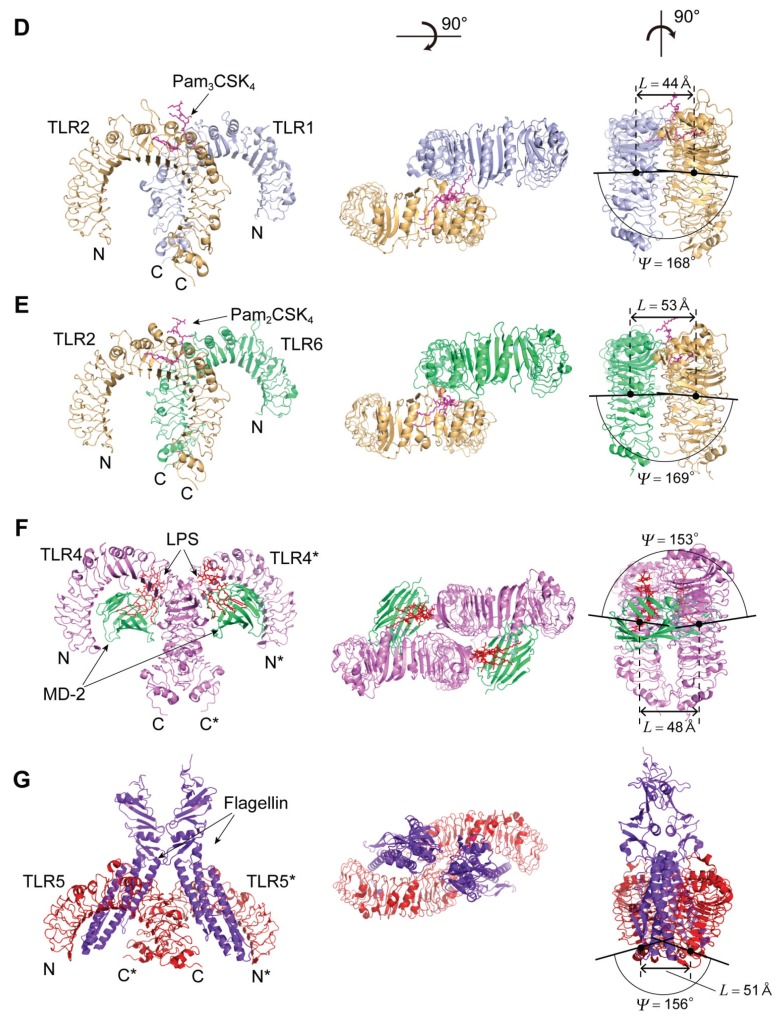
Three-dimensional schematic representation of spatial arrangement of TLR-ecto-domain dimers. (**A**) Mouse TLR3 homo-dimer in complex with dsRNA [3CIY]; (**B**) Human TLR8 homo-dimer in complex with ORN06 [4R07]; (**C**) Horse TLR9 homo-dimer in complex with agonistic DNA1668_12mer [3WPC]; (**D**) Mouse TLR1 and TLR2 hetero-dimer in complex with Pam3CSK4 [2Z7X]; (**E**) Human TLR2 and TLR6 hetero-dimer in complex with Pam2CSK4 [3A79]; (**F**) Human TLR4 homo-dimer in complex with MD-2 and LPS [3FXI]; (**G**) Zebrafish TLR5 homo-dimer in complex with flagellin [3V47]. “*L*” and “ψ” are the distance and the angle between the two helical axes of the two monomers in the dimers, respectively.

The structure of hTLR3 complexed with 46 bp dsRNA has been determined [46]. The dsRNA interacts with two TLR3-ectodomains (ECDs), forming a complex in which the two TLR3-ECDs are related by two-fold symmetry (Figure 6) [31]. The two TLR3-ECDs resemble the letter “M”, with the two N-termini extending outwards in opposite directions and the LRRCT modules converging at the center [27]. Binding occurs on the N-terminal (LRR1–LRR3) and C-terminal (LRR19–LRR21) sites on the lateral side of the convex surface of TLR3 ecto-domains, which lie on opposite sides of the dsRNA [31]. The dsRNA interacts with the ECD on the ascending lateral surface. The positively charged residues on the termini of TLR3 make major contributions to the interaction with the sugar phosphate backbones of dsRNA.

The formation of the complex induces a small decrease in the helix radii (Supplementary Table S4). The dsRNA TLR3 dimer has *L* = 74.5 Å and ψ = 148° (Figure 6 and Supplementary Table S3). The *L* value is the largest among other TLR dimers, as described later.

#### 4.2.3. The TLR3—Fab Complex

The structures of hTLR3 complexed with six different Fabs (Fab15 light and heavy chains, Fab12 light and heavy chains, and Fab1068 light and heavy chains) have been determined [45]. Fab15 binds an epitope that overlaps the C-terminal dsRNA binding site. In contrast, Fab12 and Fab1068 bind hTLR3 at sites distinct from the N-terminal and C-terminal regions that interact with dsRNA. The TLR3-Fab complex induces a decrease in *P* (Supplementary Table S4).

#### 4.2.4. The TLR8—Complexes with Agonistic Ligands

Both TLR7 and TLR8 are believed to recognize uridine and guanosine rich ssRNA as well as certain synthetic chemicals [69]. Several small molecule compounds have been identified as activators of TLR8 and TLR7 [13]. Some imidazo-quinoline derivatives, such as resiquimod (R848), are recognized by both human TLR7 and TLR8. The crystal structures of TLR8 in complex with five different agonistic chemical ligands—CL097, CL075, R848, Xg-1236, and Ds-802—have been determined (Figure 6) [53,54]. The hTLR8 structure in complex with 2-butylfuro[2,3-c]quinolin-4-amine is also available [59]. All of these complexes form liganded dimers in which the two TLR8 monomers are related by two-fold symmetry. The structure in the un-liganded dimer is also available [53]. The two chemical ligands are located in the dimer interface of TLR8 and the two positions are related by the two-fold axis. The first is located in the ascending loop close to LRR11–14 and LRR16*–18*, and the second is located in the ascending loop close to LRR11*–14* and LRR16–18 (The second TLR8 is within the dimer and its residues are indicated with asterisks).

Helix parameters of the two monomers in the un-liganded dimer are generally similar to those in the liganded dimers; although, the dimerization and/or small-molecule agonist interactions induce a small increase in *P* (Supplementary Table S4). However, the spatial arrangement of the two monomers in the dimer changes upon ligand binding. For example, the distance between the two monomers at their C-termini changes from 53 Å to 30 Å in the TLR8-CLO97 complex. The ligand binding drastically changes these two parameters; Δ*L* = −4.7 → −2.0 Å and Δψ = 10 → 17° (Supplementary Table S3).

#### 4.2.5. The TLR8—Complexes with Degradation Products of ssRNA

The hTLR8 structures in complex with its 20-mer agonist ssRNAs: ORN06, ssRNA40 and phosphorothioated ORN06 (ORN06S) have been determined [58]. TLR8 recognized two degradation products of ssRNA—uridine and a short oligonucleotide—at two distinct sites: uridine bound the site on the dimerization interface where small chemical ligands are recognized, whereas short oligonucleotides bound a newly identified site on the concave surface of the hTLR8 structure.

The hTLR8 ecto-domains form a tetramer in the crystal. The tetramer is separated into a couple of two TLR-ecto-domains. The two TLR8-ecto-domains are related by the two-fold axis as are the TLR8 complexes with antagonistic ligands. As expected, the geometrical nine parameters—*P*, Δz, ΔΦ, *N*, *R*, *p*, Ω, *L*, and ψ—are similar to those of the TLR8 complexes with antagonistic ligands (Supplementary Tables S1–S3).

#### 4.2.6. The TLR9—CpG-DNA Complex

TLR9 recognizes bacterial and viral DNA containing the cytosine phosphate guanine (CpG) dideoxynucleotide motif [70,71]. The crystal structures of three forms of TLR9: un-liganded, bound to agonistic CpG-DNA (DNA1668_12mer), and bound to inhibitory DNA (iDNA4084) have been determined [57]. Agonistic-CpG-DNA-bound TLR9 forms a symmetric TLR9—CpG-DNA complex with 2:2 stoichiometry, whereas iDNA-bound TLR9 is a monomer (Figure 6). The nine parameters of these complexes are similar to the TLR3-dsRNA complex and the TLR8 complexes with agonistic ligands and ssRNA.

The agonistic DNA binds to two equivalent positions in the dimer, and each DNA1668_12mer adopt in a bent conformation and is recognized by both TLR9 and TLR9* (The asterisk indicates the second TLR9 within the dimer) (Figure 6) [57]. DNA1668_12mer winds around the N-terminal part of TLR9 from the ascending lateral surface to the concave surface to interact with a region spanning from LRRNT to LRR10. Simultaneously, DNA1668_12mer interacts with the loop regions from LRR20*–22* in the C-terminal part of TLR9*. Thus, agonistic DNA acts as “molecular glue” to bridge the two TLR9 molecules. The iDNA binds to the concave surface from LRR2–LRR10.

### 4.3. TLR1, TLR2, and TLR6

#### The Human TLR1/2 Heterodimer and the Mouse TLR2/6 Heterodimer

TLR2 forms heterodimers with TLR1 and TLR6 [41,42]. The ligand specificity of TLR2 is modulated by changing its hetero-dimeric partners; the TLR2-TLR6 complex can bind to diacyl-lipopeptides but not to triacyl-lipopeptides. By contrast, the principal ligands of the TLR1-TLR2 complex are triacyl-lipopeptides. The interaction with diacyl-lipo-peptides is substantially weaker [6]. Pam_2_CSK_4_ and Pam_3_CSK_4_ are synthetic lipo-peptides that contain *di*- or *tri*-acylated cysteine groups at the N-termini. These peptides mimic most of the pro-inflammatory properties of lipo-proteins.

The structures of the heterodimers of hTLR1 (*n* = 20) and hTLR2 (*n* = 21), and of mTLR2 (*n* = 21) and mTLR6 (*n* = 20) complexed with Pam_3_CSK_4_ have been determined (Figure 6). The structures of the mTLR2-Pam_2_CSK_4_ and mTLR2-Pam_3_CSK_4_ complexes are also available as monomers [41,42]. The overall shape of the complex is similar to the letter “M”, as are the TLR3-dsRNA complex (Figure 6). The two N-terminal domains stretch outward at opposite ends and the C-terminal domains converge in the middle [41]. The Pam_3_CSK_4_ binding pockets of TLR1 and TLR2 are formed on the ascending lateral surface of LRRs 10–12 [29].

All of these TLRs adopt left handed helices three quarters turn long; *P* = −20.93 → −40.49 Å, Δz = −0.72 → −1.41 Å, ΔΦ = 12.33 → 12.75°, *N* = 28.23 → 29.17 units/repeat, and *R* = 22.02 → 22.96 Å (Figure 4 and Supplementary Table S1). Significantly, the LRRs in the heterodimers have similar helical parameters to each other, in comparison with those in the free monomers. The dimerization induces an increase in *P*. The values of *L* and ψ are 44.0 Å and 168° in the hTLR1/hTLR2 dimer and 53.0 Å and 169° in the mTLR2/mTLR6 dimer, respectively (Figure 6).

The structures of the mTLR2 complexes with 1,2-dimyristoyl-*sn*glycero-3-phosphoethanolamine-*N*-diethylenetriamin (PE-DTPA) and pneumococcal lipoteichoic acid (pnLTA) are also available in the monomer form [41,42]. The helical parameters of these complexes differ from one another (Supplementary Table S4). Pam_3_CSK_4_, PE-DTPA and pnLTA are located on the ascending lateral surface in the central domain of the LRRs [29].

### 4.4. TLR4

#### 4.4.1. Human and Mouse TLR4

The structure of TLR4 in complex with MD-2-LPS has been determined [48,49,50]. In addition, the structures of two antagonists of TLR4 in complex with either the TLR4-MD-2 heterodimer or with MD-2 by itself have been reported [47,56].

TLR4 (*n* = 23) forms a left handed helix of a three quarter turn as do TLR1, TLR2 and TLR6; *P* = −4.78 → −20.80 Å, Δz = −0.16 → −0.52 Å, ΔΦ = 12.00 → 12.68°, *N* = 28.40 → 30.32 units/repeat, and *R* = 22.64 → 23.98 Å (Figure 4 and Supplementary Table S1). The helix is highly distorted; *p* = 0.33 → 0.39 Å. The LRR domain consists of three subdomains. Interestingly, the helix pitch, *P*, is larger than those in TLR1, TLR2, and TLR6.

#### 4.4.2. TLR4 Complexes with MD-2/LPS

The mTLR4 and hTLR4 complexes with MD-2/LPS are available (Figure 6) [48,49,50]. The complexes with 2:2:2 stoichiometry form “M” shaped dimers in the crystal. MD-2 interacts with the concave surface of TLR4. The MD-2 binding site of TLR4 has two distinct areas, termed the A and B patches. The A patch is composed of amino acids provided by the N-terminal domain of TLR4 and is predominantly negatively charged. The B patch area is formed by residues, mostly from the central domain, most of which are positively charged. LPS is bound to the hydrophobic pocket in MD-2. LPS binding induces the formation of a symmetrical complex of two TLR4-MD-2 heterodimers [6].

These helical parameters are very similar to one another. The comparison with the monomeric mTLR4 complex with MD-2/eritoran shows that the dimerization associated with ligand interactions induces structural changes with an increase in *P* and *R* (Supplementary Table S4).

#### 4.4.3. TLR4 Complexes with Antagonistic Ligands

The mTLR4 complexes with MD-2/lipid IVa and MD-2/Eritoran are available [47,48]. These are the 2:2:2 complexes. Lipid IVa and Eritoran are lipid A derivatives with four lipid chains. Lipid IVa only confers strong antagonistic activity on human TLR4-MD-2 complex, but not the mouse complex. The mTLR4-MD-2/lipid IVa complex forms a “M” shaped dimer in the crystal. LPS, lipid IVa, and Eritoran bind to the same hydrophobic pocket of MD-2 [6]. The MD-2 pocket undergoes little structural adjustment upon binding these diverse compounds. The helical parameters are very similar to those in the TLR4-MD-2/LPS complex, as expected. Correspondingly the values of *L* and ψ are similar to each other; *L* = 47.1 → 48.3 Å and ψ =153 → 154°; the ψ’s are the smallest among the TLR dimers.

### 4.5. TLR5

#### 4.5.1. Human and Zebrafish TLR5

The ecto-domain structure of hTLR5 (*n* = 23) has been determined by electron microscopy to 26.0 Å resolution [51]. The secondary structure remains unsolved. However, the helical parameters are similar to those in TLR4. Zebrafish TLR5 contains 25 LRRs. The crystal structure of the 14 LRRs at the N-terminal side that are connected by two LRRs in hagfish VLRB.61 at the C-terminal side is available. The LRRs with *n* = 16 adopt a left-handed helix as does the entire LRR of human TLR5. The *p* value (=0.06 Å) is small and thus the helix is regular.

#### 4.5.2. TLR5-Flagellin Complex

The structure of the zebrafish TLR5 complex with a truncated fragment of *Salmonella* fliC has been determined (Figure 6) [52]. Two TLR5-ECDs interact with two flagellin molecules one on the concave surface and the other on the ascending lateral surface. The helical parameters are very similar to those of TLR5 without bound ligand (Supplementary Table S1). The values of *L* and ψ are 51 Å and 156° in the complex.

## 5. Structures of RP105, CD14, and *Drosophila* Toll Receptor

### 5.1. Human, Mouse, and Cow RP105

Two LRR proteins—RP105 (*n* = 23–24) and CD14 (*n* = 11)—are involved in TLR activation [26]. RP105 is a TLR-like, cell surface molecule that is evolutionarily closely related to TLR4 [72,73]. RP105 contains an extracellular LRR domain and transmembrane domain [74,75]. The RP105 associates with MD-1, an MD-2 homologue. Unlike TLR4, RP105 contains only ~10 intracellular residues and lacks the canonical TIR signaling domain.

**Figure 7:**
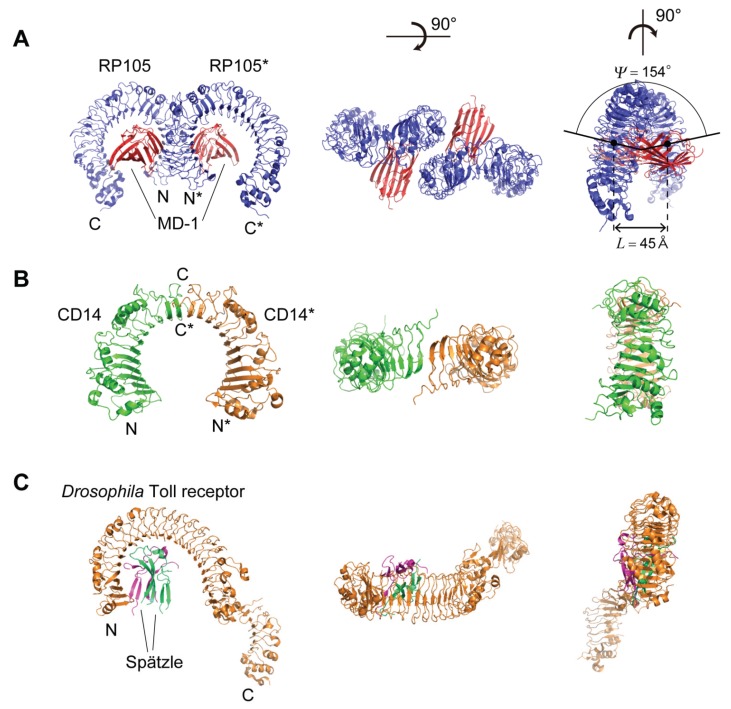
Schematic representation of the spatial arrangement of the dimers of RP105 and CD14, and the 3D structure of *Drosophila* Toll receptor. (**A**) Mouse RP105 homo-dimer in complex with MD-1 [3T6Q]; (**B**) Mouse CD14 homo-dimer [1WWL]; (**C**) *Drosophila* Toll receptor in complex with Spätzle [4LXR].

The LRRs belong to the “Typical” class as well as those in TLRs. The structures of human, mouse, and cow RP105 complexes with MD-1 have been determined [76,77]. Human and mouse RP105/MD-1 forms dimers in the 1:1 RP105/MD-1 complex (Figure 7). The “M”-shaped 2:2 RP105/MD-1 complex exhibits an inverse arrangement, with N-termini interacting in the middle (Figure 7). Thus, the dimerization interface of RP105/MD-1 is located on the opposite side of the complex, compared to the 2:2 TLR4/MD-2 complex. MD-1 interacts with not only the concave lateral surface of RP105 but also with the convex lateral surface of RP105*. Models for the interaction between RP105-D-1 and TLR4-MD-2 were proposed [77].

RP105 form left handed helices with a full turn; *P* = −4.27 → −10.09 Å, Δz = −0.16 → −0.49 Å, ΔΦ = 12.90 → 13.61°, *N* = 26.45 → 27.91 units/repeat, and *R* = 21.56 → 22.43 Å. Thus the RP105 LRR arc is approximated as an ellipse and is nearly flat. These helical parameters are comparable to those in the TLR dimers. The cow RP105 complex forms a octamer in the crystal [77]. The Ω values are larger than those in the dimers of the human and mouse complex (Supplementary Table S2), which likely reflects an effect of the crystal field.

### 5.2. Human and Mouse CD14

CD14 is a co-receptor recognizing LPS with TLR4 and MD-2 [78,79]. The structures of human and mouse CD14 are available [80,81]. A large hydrophobic pocket on the N-terminal side of CD14 contributes to LPS-binding. Mouse CD14, in which the LRR does not conform to any of the eight classes, forms a dimer in the crystallographic asymmetric unit as well as in solution (Figure 7) [81]. Dimerization in the crystal is mediated by LRR residues in the loop between β-strands in the HCS of repeats 12 and 13 [23]. The C-terminal β-strands of the two β-sheets from the two monomers interact in an anti-parallel fashion and form a large and continuous β-sheet encompassing the entire CD14 dimer. The two monomers are related by a two-fold axis of rotation, perpendicular to their common helix axis. The entire dimer forms a left handed helix, as does the CD14 monomer; *P* = −32.31 Å, Δz = −1.12 Å, ΔΦ = 12.47°, *N* = 28.86 units/repeat, and *R* = 22.59 Å [23].

In addition to the TLRs, RP105, and CD14, left-handed helices are also observed in FSHR, Lmof2365_1307 and LEGL7 with *n* ≥ 7 [23]. In their LRRs the HCS parts in several or some repeats do not adopt short β-strands but long β-strands except for LEG7. It appears that the long β-strands are an important factor to induce a change in the handedness.

### 5.3. Drosophila Toll Receptor

The crystal structure of a 1:1 (non-signaling) complex of the *Drosophila* Toll receptor ecto-domain with the Spätzle cystine knot domain dimer is available (Figure 7) [82]. The LRRs (*n* = 24) belong to the “Typical“ class and form two subdomains, LRRs 1–19 and LRRs 20–24 [23]. In contrast to the left-handed helices in TLR1-2, TLR4-5, and TLR6, the first subdomain adopts a right-handed helix; *P* = 40.25 → 41.60 Å, Δz = 1.14 → 1.20 Å, ΔΦ = 10.23 → 10.35°, *N* = 34.77 → 35.19 units/repeat, and *R* = 26.64 → 27.12 Å [23]. The Spätzle ligand interacts with the concave face of the first subdomain.

## 6. Diversity of Protein Protein/Ligand Interactions

In the LRRs the concave surface, the convex surface, N-terminal domain including N-cap, C-terminal domain including C-cap, or their combinations are involved in protein, protein interactions [23]. TLRs frequently use the ascending lateral surfaces, but not the descending lateral surface, less frequently the concave face, and rarely the convex face for various interactions. Moreover, the dimerizations of TLRs are essential for these interactions. A diversity of the ascending loop conformation and dimerizations enables interactions with various molecules including lipids, RNAs, DNAs, and proteins.

## 7. Conclusions

From this review several conclusions emerge about the LRR structures in NLRs and TLRs.

(1) The LRRs in NLRs and TLRs belong to the “RI-like” and “Typical” class, respectively. The LRR units of the two classes show secondary structure motifs characteristic of the class.
(2) The nine geometrical parameters—*P*, Δz, ΔΦ, *N*, *R*, *p*, Ω, *L*, and ψ—characterize the LRR structures in NLRs and TLRs. The LRRs in NLRC4, NLRP1, and NLRX1 adopt a right-handed helix, while TLR1-5, TLR6, TLR8, and TLR9 adopt either a left-handed helix or are nearly flat. Moreover, RP105 and CD14 also adopt a left-handed helix.
(3) The helical parameters of TLRs are classified in four groups; they are TLR3/TLR8/9, TLR1/2/6, TLR4, and TLR5. This is closely related to the phylogenetic tree based on the amino acids sequences [60,61,62,63].
(4) The LRRs in NLRC4 and plant TIR1/COI1 adopt a distorted right-handed helix with one full turn. All or most of the individual LRR units adopt a β-α structural motif.
(5) Consequently, the spatial arrangement of the NACHT domain in mNLRC4 is similar to that of SKP1A in the AtTIR1/AtCOI1 complex.
(6) In the TLRs the ascending lateral surface are involved in protein, protein/ligand interactions. The diversity of the ascending loop conformations and of the dimerization conformations enables interactions with various targets that range from small molecules to proteins.

## Supplementary Files

Supplementary File 1

## References

[1] Motta V., Soares F., Sun T., Philpott D.J. (2015). NOD-like receptors: Versatile cytosolic sentinels. Physiol. Rev..

[2] Lupfer C., Kanneganti T.D. (2013). Unsolved mysteries in NLR biology. Front. Immunol..

[3] Oviedo-Boyso J., Bravo-Patiño A., Baizabal-Aguirre V. (2014). Collaborative action of Toll-like and NOD-like receptors as modulators of the inflammatory response to pathogenic bacteria. Mediat. Inflamm..

[4] Yao Q. (2013). Nucleotide-binding oligomerization domain containing 2: Structure, function, and diseases. Semin. Arthritis Rheum..

[5] Pandey S., Kawai T., Akira S. (2015). Microbial sensing by Toll-like receptors and intracellular nucleic acid sensors. Cold Spring Harb. Perspect. Med..

[6] Kang J.Y., Lee J.O. (2011). Structural biology of the Toll-like receptor family. Annu. Rev. Biochem..

[7] Robinson J., Moehle K. (2014). Structural aspects of molecular recognition in the immune system. Part II: Pattern recognition receptors. Pure Appl. Chem..

[8] Berglund N., Kargas V., Ortiz-Suarez M.L., Bond P.J. (2015). The role of protein-protein interactions in Toll-like receptor function. Prog. Biophys. Mol. Biol..

[9] Gay N.J., Gangloff M. (2007). Structure and function of Toll receptors and their ligands. Annu. Rev. Biochem..

[10] Weber A.N., Tauszig-Delamasure S., Hoffmann J.A., Lelievre E., Gascan H., Ray K.P., Morse M.A., Imler J.L., Gay N.J. (2003). Binding of the *Drosophila* cytokine Spatzle to Toll is direct and establishes signaling. Nat. Immunol..

[11] Lemaitre B., Nicolas E., Michaut L., Reichhart J.M., Hoffmann J.A. (1996). The dorsoventral regulatory gene cassette spatzle/Toll/cactus controls the potent antifungal response in *Drosophila* adults. Cell.

[12] Zhang J., Kong X., Zhou C., Li L., Nie G., Li X. (2014). Toll-like receptor recognition of bacteria in fish: Ligand specificity and signal pathways. Fish Shellfish Immunol..

[13] Hennessy E.J., Parker A.E., O’Neill L.A. (2010). Targeting Toll-like receptors: Emerging therapeutics?. Nat. Rev. Drug Discov..

[14] Kobe B., Deisenhofer J. (1994). The leucine-rich repeat: A versatile binding motif. Trends Biochem. Sci..

[15] Kobe B., Kajava A.V. (2001). The leucine-rich repeat as a protein recognition motif. Curr. Opin. Struct. Biol..

[16] Matsushima N., Enkhbayar P., Kamiya M., Osaki M., Kretsinger R. (2005). Leucine-Rich Repeats (LRRs): Structure, Function, Evolution and Interaction with Ligands. Drug Des. Rev..

[17] Matsushima N., Tachi N., Kuroki Y., Enkhbayar P., Osaki M., Kamiya M., Kretsinger R.H. (2005). Structural analysis of leucine-rich-repeat variants in proteins associated with human diseases. Cell. Mol. Life Sci..

[18] Bella J., Hindle K.L., McEwan P.A., Lovell S.C. (2008). The leucine-rich repeat structure. Cell. Mol. Life Sci..

[19] Enkhbayar P., Kamiya M., Osaki M., Matsumoto T., Matsushima N. (2004). Structural principles of leucine-rich repeat (LRR) proteins. Proteins.

[20] Kajava A.V., Anisimova M., Peeters N. (2008). Origin and evolution of GALA-LRR, a new member of the CC-LRR subfamily: From plants to bacteria?. PLoS ONE.

[21] Kajava A.V. (1998). Structural diversity of leucine-rich repeat proteins. J. Mol. Biol..

[22] Matsushima N., Miyashita H., Mikami T., Kuroki Y. (2010). A nested leucine rich repeat (LRR) domain: The precursor of LRRs is a ten or eleven residue motif. BMC Microbiol..

[23] Enkhbayar P., Miyashita H., Kretsinger R., Matsushima N. (2014). Helical parameters and correlations of tandem leucine rich repeats in proteins. J. Proteom. Bioinform..

[24] Enkhbayar P., Damdinsuren S., Osaki M., Matsushima N. (2008). HELFIT: Helix fitting by a total least squares method. Comput. Biol. Chem..

[25] Enkhbayar P., Matsushima N. (2012). HELFIT: Algorithm and applications. AIP Conf. Proc..

[26] Song D.H., Lee J.O. (2012). Sensing of microbial molecular patterns by Toll-like receptors. Immunol. Rev..

[27] Jin M.S., Lee J.O. (2008). Structures of the toll-like receptor family and its ligand complexes. Immunity.

[28] Jin M.S., Lee J.O. (2008). Structures of TLR-ligand complexes. Curr. Opin. Immunol..

[29] Botos I., Segal D.M., Davies D.R. (2011). The structural biology of Toll-like receptors. Structure.

[30] Carpenter S., O’Neill L.A. (2009). Recent insights into the structure of Toll-like receptors and post-translational modifications of their associated signalling proteins. Biochem. J..

[31] Botos I., Liu L., Wang Y., Segal D.M., Davies D.R. (2009). The toll-like receptor 3:dsRNA signaling complex. Biochim. Biophys. Acta.

[32] Lechtenberg B.C., Mace P.D., Riedl S.J. (2014). Structural mechanisms in NLR inflammasome signaling. Curr. Opin. Struct. Biol..

[33] ccPDB. http://crdd.osdd.net/raghava/ccpdb/beta2_up.php.

[34] Reubold T.F., Hahne G., Wohlgemuth S., Eschenburg S. (2014). Crystal structure of the leucine-rich repeat domain of the NOD-like receptor NLRP1: Implications for binding of muramyl dipeptide. FEBS Lett..

[35] Hong M., Yoon S.I., Wilson I.A. (2012). Structure and functional characterization of the RNA-binding element of the NLRX1 innate immune modulator. Immunity.

[36] Hu Z., Yan C., Liu P., Huang Z., Ma R., Zhang C., Wang R., Zhang Y., Martinon F., Miao D. (2013). Crystal structure of NLRC4 reveals its autoinhibition mechanism. Science.

[37] Faustin B., Lartigue L., Bruey J.M., Luciano F., Sergienko E., Bailly-Maitre B., Volkmann N., Hanein D., Rouiller I., Reed J.C. (2007). Reconstituted NALP1 inflammasome reveals two-step mechanism of caspase-1 activation. Mol. Cell.

[38] Tan X., Calderon-Villalobos L.I., Sharon M., Zheng C., Robinson C.V., Estelle M., Zheng N. (2007). Mechanism of auxin perception by the TIR1 ubiquitin ligase. Nature.

[39] Hayashi K., Tan X., Zheng N., Hatate T., Kimura Y., Kepinski S., Nozaki H. (2008). Small-molecule agonists and antagonists of F-box protein-substrate interactions in auxin perception and signaling. Proc. Natl. Acad. Sci. USA.

[40] Sheard L.B., Tan X., Mao H., Withers J., Ben-Nissan G., Hinds T.R., Kobayashi Y., Hsu F.F., Sharon M., Browse J. (2010). Jasmonate perception by inositol-phosphate-potentiated COI1-JAZ co-receptor. Nature.

[41] Jin M.S., Kim S.E., Heo J.Y., Lee M.E., Kim H.M., Paik S.G., Lee H.Y., Lee J.O. (2007). Crystal structure of the TLR1-TLR2 heterodimer induced by binding of a tri-acylated lipopeptide. Cell.

[42] Kang J.Y., Nan X., Jin M.S., Youn S.J., Ryu Y.H., Mah S., Han S.H., Lee H., Paik S.G., Lee J.O. (2009). Recognition of Lipopeptide Patterns by Toll-like Receptor 2-Toll-like Receptor 6 Heterodimer. Immunity.

[43] Choe J., Kelker M.S., Wilson I.A. (2005). Crystal structure of human Toll-like receptor 3 (TLR3) ectodomain. Science.

[44] Bell J.K., Botos I., Hall P.R., Askins J., Shiloach J., Segal D.M., Davies D.R. (2005). The molecular structure of the Toll-like receptor 3 ligand-binding domain. Proc. Natl. Acad. Sci. USA.

[45] Luo J.Q., Obmolova G., Malia T.J., Wu S.J., Duffy K.E., Marion J.D., Bell J.K., Ge P., Zhou Z.H., Teplyakov A. (2012). Lateral clustering of TLR3:dsRNA signaling units revealed by TLR3ecd:3Fabs quaternary structure. J. Mol. Biol..

[46] Liu L., Botos I., Wang Y., Leonard J.N., Shiloach J., Segal D.M., Davies D.R. (2008). Structural basis of toll-like receptor 3 signaling with double-stranded RNA. Science.

[47] Kim H.M., Park B.S., Kim J.I., Kim S.E., Lee J., Oh S.C., Enkhbayar P., Matsushima N., Lee H., Yoo O.J. (2007). Crystal structure of the TLR4-MD-2 complex with bound endotoxin antagonist Eritoran. Cell.

[48] Ohto U., Fukase K., Miyake K., Shimizu T. (2012). Structural basis of species-specific endotoxin sensing by innate immune receptor TLR4/MD-2. Proc. Natl. Acad. Sci. USA.

[49] Park B.S., Song D.H., Kim H.M., Choi B.S., Lee H., Lee J.O. (2009). The structural basis of lipopolysaccharide recognition by the TLR4-MD-2 complex. Nature.

[50] Ohto U., Yamakawa N., Akashi-Takamura S., Miyake K., Shimizu T. (2012). Structural Analyses of Human Toll-like Receptor 4 Polymorphisms D299G and T399I. J. Biol. Chem..

[51] Zhou K.F., Kanai R., Lee P., Wang H.W., Modis Y. (2012). Toll-like receptor 5 forms asymmetric dimers in the absence of flagellin. J. Struct. Biol..

[52] Yoon S.I., Kurnasov O., Natarajan V., Hong M.S., Gudkov A.V., Osterman A.L., Wilson I.A. (2012). Structural Basis of TLR5-Flagellin Recognition and Signaling. Science.

[53] Tanji H., Ohto U., Shibata T., Miyake K., Shimizu T. (2013). Structural Reorganization of the Toll-Like Receptor 8 Dimer Induced by Agonistic Ligands. Science.

[54] Yoo E., Salunke D., Sil D., Guo X., Salyer A., Hermanson A., Kumar M., Malladi S., Balakrishna R., Thompson W. (2014). Determinants of activity at human Toll-like receptors 7 and 8: Quantitative structure-activity relationship (QSAR) of diverse heterocyclic scaffolds. J. Med. Chem..

[55] Collins B., Wilson I.A. (2014). Crystal structure of the C-terminal domain of mouse TLR9. Proteins.

[56] Ohto U., Fukase K., Miyake K., Satow Y. (2007). Crystal structures of human MD-2 and its complex with antiendotoxic lipid IVa. Science.

[57] Ohto U., Shibata T., Tanji H., Ishida H., Krayukhina E., Uchiyama S., Miyake K., Shimizu T. (2015). Structural basis of CpG and inhibitory DNA recognition by Toll-like receptor 9. Nature.

[58] Tanji H., Ohto U., Shibata T., Taoka M., Yamauchi Y., Isobe T., Miyake K., Shimizu T. (2015). Toll-like receptor 8 senses degradation products of single-stranded RNA. Nat. Struct. Mol. Biol..

[59] Kokatla H.P., Sil D., Tanji H., Ohto U., Malladi S.S., Fox L.M., Shimizu T., David S.A. (2014). Structure-based design of novel human Toll-like receptor 8 agonists. ChemMedChem.

[60] Matsushima N., Tanaka T., Enkhbayar P., Mikami T., Taga M., Yamada K., Kuroki Y. (2007). Comparative sequence analysis of leucine-rich repeats (LRRs) within vertebrate toll-like receptors. BMC Genom..

[61] Roach J.C., Glusman G., Rowen L., Kaur A., Purcell M.K., Smith K.D., Hood L.E., Aderem A. (2005). The evolution of vertebrate Toll-like receptors. Proc. Natl. Acad. Sci. USA.

[62] Zhou H., Gu J., Lamont S.J., Gu X. (2007). Evolutionary analysis for functional divergence of the toll-like receptor gene family and altered functional constraints. J. Mol. Evol..

[63] Mikami T., Miyashita H., Takatsuka S., Kuroki Y., Matsushima N. (2012). Molecular evolution of vertebrate Toll-like receptors: Evolutionary rate difference between their leucine-rich repeats and their TIR domains. Gene.

[64] Matsushima N., Ohyanagi T., Tanaka T., Kretsinger R.H. (2000). Super-motifs and evolution of tandem leucine-rich repeats within the small proteoglycans—Biglycan, decorin, lumican, fibromodulin, PRELP, keratocan, osteoadherin, epiphycan, and osteoglycin. Proteins.

[65] Matsushima N., Kamiya M., Suzuki N., Tanaka T. (2000). Super-motifs of leucine-rich repeats (LRRs) proteins. Genome Inform..

[66] Scott P.G., Dodd C.M., Bergmann E.M., Sheehan J.K., Bishop P.N. (2006). Crystal structure of the biglycan dimer and evidence that dimerization is essential for folding and stability of class I small leucine-rich repeat proteoglycans. J. Biol. Chem..

[67] Scott P.G., McEwan P.A., Dodd C.M., Bergmann E.M., Bishop P.N., Bella J. (2004). Crystal structure of the dimeric protein core of decorin, the archetypal small leucine-rich repeat proteoglycan. Proc. Natl. Acad. Sci. USA.

[68] Seiradake E., del Toro D., Nagel D., Cop F., Hartl R., Ruff T., Seyit-Bremer G., Harlos K., Border E.C., Acker-Palmer A. (2014). FLRT structure: Balancing repulsion and cell adhesion in cortical and vascular development. Neuron.

[69] Heil F., Hemmi H., Hochrein H., Ampenberger F., Kirschning C., Akira S., Lipford G., Wagner H., Bauer S. (2004). Species-specific recognition of single-stranded RNA via toll-like receptor 7 and 8. Science.

[70] Bauer S., Kirschning C.J., Hacker H., Redecke V., Hausmann S., Akira S., Wagner H., Lipford G.B. (2001). Human TLR9 confers responsiveness to bacterial DNA via species-specific CpG motif recognition. Proc. Natl. Acad. Sci. USA.

[71] Hemmi H., Takeuchi O., Kawai T., Kaisho T., Sato S., Sanjo H., Matsumoto M., Hoshino K., Wagner H., Takeda K. (2000). A Toll-like receptor recognizes bacterial DNA. Nature.

[72] Miyake K., Yamashita Y., Ogata M., Sudo T., Kimoto M. (1995). RP105, a novel B cell surface molecule implicated in B cell activation, is a member of the leucine-rich repeat protein family. J. Immunol..

[73] Divanovic S., Trompette A., Atabani S.F., Madan R., Golenbock D.T., Visintin A., Finberg R.W., Tarakhovsky A., Vogel S.N., Belkaid Y. (2005). Negative regulation of Toll-like receptor 4 signaling by the Toll-like receptor homolog RP105. Nat. Immunol..

[74] Miyake K., Shimazu R., Kondo J., Niki T., Akashi S., Ogata H., Yamashita Y., Miura Y., Kimoto M. (1998). Mouse MD-1, a molecule that is physically associated with RP105 and positively regulates its expression. J. Immunol..

[75] Inohara N., Nunez G. (2002). ML—A conserved domain involved in innate immunity and lipid metabolism. Trends Biochem. Sci..

[76] Ohto U., Miyake K., Shimizu T. (2011). Crystal Structures of Mouse and Human RP105/MD-1 Complexes Reveal Unique Dimer Organization of the Toll-Like Receptor Family. J. Mol. Biol..

[77] Yoon S.I., Hong M.S., Wilson I.A. (2011). An unusual dimeric structure and assembly for TLR4 regulator RP105-MD-1. Nat. Struct. Mol. Biol..

[78] Fujihara M., Muroi M., Tanamoto K., Suzuki T., Azuma H., Ikeda H. (2003). Molecular mechanisms of macrophage activation and deactivation by lipopolysaccharide: Roles of the receptor complex. Pharmacol. Ther..

[79] Miyake K. (2003). Innate recognition of lipopolysaccharide by CD14 and toll-like receptor 4-MD-2: Unique roles for MD-2. Int. Immunopharmacol..

[80] Kelley S.L., Lukk T., Nair S.K., Tapping R.I. (2013). The Crystal Structure of Human Soluble CD14 Reveals a Bent Solenoid with a Hydrophobic Amino-Terminal Pocket. J. Immunol..

[81] Kim J.I., Lee C.J., Jin M.S., Lee C.H., Paik S.G., Lee H., Lee J.O. (2005). Crystal structure of CD14 and its implications for lipopolysaccharide signaling. J. Biol. Chem..

[82] Parthier C., Stelter M., Ursel C., Fandrich U., Lilie H., Breithaupt C., Stubbs M.T. (2014). Structure of the Toll-Spatzle complex, a molecular hub in *Drosophila* development and innate immunity. Proc. Natl. Acad. Sci. USA.

