# Supplementary material for: Comparative Geometrical Analysis of Leucine-Rich Repeat Structures in the Nod-Like and Toll-Like Receptors in Vertebrate Innate Immunity
Source: Biomolecules. 2015 Aug 18;5(3):1955–78. doi: 10.3390/biom5031955 (PMC4598782; doi:10.3390/biom5031955)
Supplement: Supplementary File 1 [file biomolecules-05-01955-s001.zip › biomolecules-94696-supplementary-publish/Suppl_Figure_S1.docx]

**Supplementary Figure S1.** Secondary structure. Yellow color indicates β-strands; Red color indicates β-turns; Green color indicates α-helix; Light blue color indicates 3_10_-helix.

>4IM6_A

GAMGGSPV TDAYWQILFSVLKVTRN

LKELDLSGNSL SHSAVKSLCKTLRRPRCL

LETLRLAGCGL TAEDCKDLAFGLRANQT

LTELDLSFNVL TDAGAKHLCQRLRQPSCK

LQRLQLVSCGL TSDCCQDLASVLSASPS

LKELDLQQNNL DDVGVRLLCEGLRHPACK

LIRLGLDQTTL SDEMRQELRALEQEKP

QLLIFSRRKPS

>3UN9_A

>NLRX1_HUMAN

MLLVGLLSAHNRAVLAQLGCPIKNLDALENAQAIKKKLGKLGRQVLP

PSELLDHLFFHYEFQNQRFSAEVLSS

LRQLNLAGVRM TPVKCTVVAAVLGSGRHA

LDEVNLASCQL DPAGLRTLLPVFLR

ARKLGLQLNSL GPEACKDLRDLLLHDQCQ

ITTLRLSNNPL TAAGVAVLMEGLAGNTS

VTHLSLLHTGL GDEGLELLAAQLDRNRQ

LQELNVAYNGA GDTAALALARAAREHPS

LELLHLYFNEL SSEGRQVLRD**LGGAAEG**

GARVVVSLTEG TAVSEYWSVILSEVQRN

LNSWDRARVQRHLELLLRDLEDSRGATLNP

WRKAQLLRVEGEVRALLEQLGSSGSPSGSWSHPQFEKGAGHHHHHH

>4KXF

>NLRC4_MOUSE

MNFIRNNRRALIQRMGLTVTKQICDDLFALNVLNNQEANVIYCEPLEQEAARKIIHMTM

QKGSAACNLFLKSLENWDYFVYQDLTGQNLSYQVTEEDLNVLAQNLKDLYNSPAFLNFY

PLGEDIDIIFNLEKTFTEPIMWKKDHRHHRVEQLTLGSLLEALKSPCLIEGESGKGKST

LLQRIAMLWASGGCRALKGFRLVFFIHLRSARGGLFETLYDQLLNIPDFISKPTFKALL

LKLHKEVLFLLDGYNEFHPQNCPEIEALIKENHRFKNMVIVTTTTECLRHIRHVGALTA

EVGDMTEDSAKDLIEAVLVPDQVERLWAQIQESRCLRNLMKTPLFVVITCAIQMGRQEF

QAHTQTMLFQTFYDLLIQKNSHRYRGGASGDFARSLDYCGDLALEGVFAHKFDFEPEHG

SSMNEDVLVTIGLLCKYTAQRLKPTYKFFHKSFQEYTAGRRLSSLLTSKEPEEVSKGNS

YLNKMVSISDITSLYGNLLLYTCGSSTEATRAVMRHLAMVYQHGSLQGLSVTKRPLWRQ

ESIQSLRNTTEQDVLKAINVNSFVECGINLFSESMSKSDLSQEFEAFFQ

GKSLYINSENIPDYL FDFFEYLPNCASALD

FVKLDFYERAT**ES QDKAEENVPGVHTEGPSETYI**PPRAVSLFFNWKQEFK

TLEVTLRDINKL NKQDIKYLGKIFSSAT

NLRLHIKRCAAM AGRLSSVLRTCKN

MHTLMVEASPL TTDDEQYITSVTG

LQNLSIHRLHT QQLPGGLIDSLGNLKN

LERLILDDIRM NEEDAKNLAEGLRSLKK

MRLLHLTHLSDI GEGMDYIVKSLSEESCD

LQEMKLVACCL TANSVKVLAQNLHNLIK

LSILDISENYL EKDGNEALQELIGRLGVLGE

LTTLMLPWCWDV HTSLPKLLKQLEGTPG

LAKLGLKNWRL RDEEIKSLGEFLEMNPLRD

LQQLDLAGHCV SSDGWLYFMNVFENLKQ

LVFFDFSTEEFL PDAALVRKLSQVLSKLTL

LQEVKLTGWEF DDYDISAIKG

TFKLVTA

>4PER_A

>Chicken RI

YFQ

GMDLDIQCEEI NPSRWAELLSTMKS

CSTIRLDDCNL SSSNCKDLSSIIHTNPS

LKELKLNNNEL GDAGIEYLCKGLLTPSCS

LQKLWLQNCNL TSASCETLRSVLSAQPS

LTELHVGDNKL GTAGVKVLCQGLMNPNCK

LQKLQLEYCEL TADIVEALNAALQAKPT

LKELSLSNNTL GDTAVKQLCRGLVEASCD

LELLHLENCGI TSDSCRDISAVLSSKPS

LLDLAVGDNKI GDTGLALLCQGLLHPNCK

IQKLWLWDCDL TSASCKDLSRVFSTKET

LLEVSLIDNNL RDSGMEMLCQALKDPKAH

LQELWVRECGL TAACCKAVSSVLSVNKH

LQVLHIGENKL GNAGVEILCEGLLHPNCN

IHSLWLGNCDI TAACCATLANVMVTKQN

LTELDLSYNTL EDEGVMKLCEAVRNPNCK

MQQLILYDIFWGPEVDDELKALEEARP

DVKIIS

>3CIG_A

>Mouse TLR3

QCTVR

YNVADCSHLKL THIPDDLPSN

ITVLNLTHNQL RRLPPTNFTRYSQ

LAILDAGFNSI SKLEPELCQILPL

LKVLNLQHNEL SQISDQTFVFCTN

LTELDLMSNSI HKIKSNPFKNQKN

LIKLDLSHNGL SSTKLGTGVQLEN

LQELLLAKNKI LALRSEELEFLGNSS

LRKLDLSSNPL KEFSPGCFQTIGK

LFALLLNNAQL NPHLTEKLCWELSNTS

IQNLSLANNQL LATSESTFSGLKWTN

LTQLDLSYNNL HDVGNGSFSYLPS

LRYLSLEYNNI QRLSPRSFYGLSN

LRYLSLKRAFTKQSVSLASHPNIDDFSFQWLKY

LEYLNMDDNNI PSTKSNTFTGLVS

LKYLSLSKTFTSL QTLTNETFVSLAHSP

LLTLNLTKNHI SKIANGTFSWLGQ

LRILDLGLNEI EQKLSGQEWRGLRN

IFEIYLSYNKYL QLSTSSFALVPS

LQRLMLRRVAL KNVDISPSPFRPLRN

LTILDLSNNNI ANINEDLLEGLEN

LEILDFQHNNLARLW**KRANPGGP**VNFLKGLSH

LHILNLESNGL DEIPVGVFKNLFE

LKSINLGLNNL NKLEPFIFDDQTS

LRSLNLQKNLI TSVEKDVFGPPFQN

LNSLDMRFNPF DCTCESISWFVNWINQTHT

NISELSTHYLCNTPHHYYGFPLKLFDTSSCKDSAPFENLYFQGHHHHHHWSHPQFEK

>1ZIW_A

>Human TLR3

KCTVS

HEVADCSHLKL TQVPDDLPTN

ITVLNLTHNQL RRLPAANFTRYSQ

LTSLDVGFNTI SKLEPELCQKLPM

LKVLNLQHNEL SQLSDKTFAFCTN

LTELHLMSNSI QKIKNNPFVKQKN

LITLDLSHNGL SSTKLGTQVQLEN

LQELLLSNNKI 　 QALKSEELDIFANSS

LKKLELSSNQI 　 KEFSPGCFHAIGR

LFGLFLNNVQL GPSLTEKLCLELANTS

IRNLSLSNSQL STTSNTTFLGLKWTN

LTMLDLSYNNL NVVGNDSFAWLPQ

LEYFFLEYNNI QHLFSHSLHGLFN

VRYLNLKRSFTKQ**SISLAS**LPKIDDFSFQWLKC

LEHLNMEDNDI PGIKSNMFTGLIN

LKYLSLSNSFT SLRTLTNETFVSLAHSP

LHILNLTKNKI SKIESDAFSWLGH

LEVLDLGLNEI GQELTGQEWRGLEN

IFEIYLSYNKY LQLTRNSFALVPS

LQRLMLRRVAL KNVDSSPSPFQPLRN

LTILDLSNNNI ANINDDMLEGLEK

LEILDLQHNNLARLWKHANPGGPIYFLKGLSH

LHILNLESNGF DEIPVEVFKDLFE

LKIIDLGLNNL NTLPASVFNNQVS

LKSLNLQKNLI TSVEKKVFGPAFRN

LTELDMRFNPF DCTCESIAWFVNWINET

HTNIPELSSHYLCNTPPHYHGFPVRLFDTSSCKDSAHHHHHH

>3W3J_A

>Human TLR8

RSPWEENFSRSYPCDEKKQND

SVIAECSNRRL QEVPQTVGKY

VTELDLSDNFI THITNESFQGLQN

LTKINLNHNPNV**QHQNGNPGIQSN**GLNITDGAFLNLKN

LRELLLEDNQL PQIPSGLPES

LTELSLIQNNI YNITKEGISRLIN

LKNLYLAWNCYFNKVCEK TNIEDGVFETLTN

LELLSLSFNSL SHVPPKLPSS

LRKLFLSNTQI KYISEEDFKGLIN

LTLLDLSGNCPRCFNAPFPCVPCDGGASINIDRFAFQNLTQ

LRYLNLSSTSL RKINAAWFKNMPH

LKVLDLEFNYLV GEIASGAFLTMLPR

LEILDLSFNYI KGSYPQHINISRNFSKLLS

LRALHLRGYVF QELREDDFQPLMQLPN

LSTINLGINFI KQIDFKLFQNFSN

LEIIYLSENRISP**LVKDTRQSYANSSSFQRHIRKRRST**DFEFDPHSN

FYHFTRPLIKPQCAAY

GKALDLSLNSI FFIGPNQFENLPD

IACLNLSANSNA QVLSGTEFSAIPH

VKYLDLTNNRL DFDNASALTELSD

LEVLDLSYNSHYFRI AGVTHHLEFIQNFTN

LKVLNLSHNNI YTLTDKYNLESKS

LVELVFSGNRL DILWNDDDNRYISIFKGLKN

LTRLDLSLNRL KHIPNEAFLNLPAS

LTELHINDNML KFFNWTLLQQFPR

LELLDLRGNKL LFLTDSLSDFTSS

LRTLLLSHNRI SHLPSGFLSEVSS

LKHLDLSSNLL KTINKSALETKTTTK

LSMLELHGNPFECTCDIGDFRRWMDEHLNVKIPRLVDVICASPGDQRGKSIVSLELTTCVSDVTEFLVPR

>3WPC_A

>Horse TLR9

RSPWQGTLPPFLPCELQP

HGLVNCNWLFL KSVPHFSAAAPRDN

VTSLSLLSNRI HHLHDSDFAQLSN

LQKLNLKWNCPPAGLSPMHFPCH MTIEPNTFLAVPT

LEELNLSYNGI TTVPALPSS

LVSLILSRTNI 　 LQLDPTSLTGLHA

LRFLYMDGNCYYKNPCGRA 　　LEVAPGALLGLGN

LTHLSLKYNNL TTVPRSLPPS

LEYLLLSYNHI VTLAPEDLANLTA

LRVLDVGGNCRRCDHARNPCVECPHKFPQLHSDTFSHLSR

LEGLVLKDSSL YQLNPRWFRGLGN

LTVLDLSENFL YDCITKTKAFQGLAQ

LRRLNLSFNYHKKV SFAHLTLAPSFGSLLS

LQELDMHGIFF RSLSQKTLQPLARLPM

LQRLYLQMNFI NQAQLGIFKDFPG

LRYIDLSDNRISGAVE**PVATTGEVDGGKKVWLTSRDLTPGPLDTPSS**EDFMPSCKNL

SFTLDLSRNNL VTVQPEMFAQLSR

LQCLRLSHNSI SQAVNGSQFVPLTS

LQVLDLSHNKL DLYHGRSFTELPR

LEALDLSYNSQPF SMRGVGHNLSFVAQLPT

LRYLSLAHNGI HSRVSQQLCSTS

LWALDFSGNSL SQMWAEGDLYLRFFQGLRS

LIRLDLSQNRL HTLLPCTLGNLPKS

LQLLRLRNNYL AFFNWSSLTLLPN

LETLDLAGNQL KALSNGSLPSGTQ

LQRLDVSRNSI IFVVPGFFALATR

LRELNLSANAL RTVEPSWFGFLAGS

LEVLDVSANPL HCACGAAFVDFLLQ

VQAAVPGLPSRVKCGSPGQLQGRSIFAQDLRLCLDESLSWDEFLVPR

>3WPE_A

>Bovine TLR9

RSPWEGTL

PAFLPCELQP

HGQVDCNWLFL KSVPHFSAGAPRAN

VTSLSLISNRI HHLHDSDFVHLSN

LRVLNLKWNCPPAGLSPMHFPCR MTIEPNTFLAVPT

LEELNLSYNGI TTVPALPSS

LVSLSLSHTSI LVLGPTHFTGLHA

LRFLYMDGNCY YMNPCPRALEVAPGALLGLGN

LTHLSLKYNNL TEVPRRLPPS

LDTLLLSYNHI VTLAPEDLANLTA

LRVLDVGGNCRRCDHARNPCRECPKNFPKLHPDTFSHLSR

LEGLVLKDSSL YKLEKDWFRGLGR

LQVLDLSENFL YDYITKTTIFNDLTQ

LRRLNLSFNYHKKV SFAHLHLASSFGSLVS

LEKLDMHGIFF RSLTNITLQSLTRLPK

LQSLHLQLNFI NQAQLSIFGAFPS

LLFVDLSDNRIS**GAATPAAALGEVDSRVEVWRLPRGLAPGPLDAVSSKDFMP**SCNL

NFTLDLSRNNL VTIQQEMFTRLSR

LQCLRLSHNSI SQAVNGSQFVPLTS

LRVLDLSHNKL DLYHGRSFTELPQ

LEALDLSYNSQPF SMQGVGHNLSFVAQLPS

LRYLSLAHNGI HSRVSQKLSSAS

LRALDFSGNSL SQMWAEGDLYLCFFKGLRN

LVQLDLSENHL HTLLPRHLDNLPKS

LRQLRLRDNNL AFFNWSSLTVLPR

LEALDLAGNQL KALSNGSLPPGIR

LQKLDVSSNSI GFVIPGFFVRATR

LIELNLSANAL KTVDPSWFGSLAGT

LKILDVSANPL HCACGAAFVDFLLERQEA

VPGLSRRVTCGSPGQLQGRSIFTQDLRLCLDETLSLDEFLVPR

>3WPF_A

>Mouse TLR9

>27 repeats

RSPWLGTLPAFLPCELKP

HGLVDCNWLFL KSVPRFSAAASCSN

ITRLSLISNRI HHLHNSDFVHLSN

LRQLNLKWNCPPTGLS**PLHF**SCH MTIEPRTFLAMRT

LEELNLSYNGI TTVPRLPSS

LVNLSLSHTNI LVLDANSLAGLYS

LRVLFMDGNCYYKNPCTGA VKVTPGALLGLSQ

LTHLSLKYNNL TKVPRQLPPS

LEYLLVSYNLI VKLGPEDLAQLTS

LRVLDVGGNCRRCDHAPNPCIECGQKSLHLHPETFHHLSH

LEGLVLKDSSL HTLNSSWFQGLVQ

LSVLDLSENFL YESITHTNAFQNLTR

LRKLNLSFNYRKKV SFARLHLASSFKNLVS

LQELNMNGIFF RLLNKYTLRWLADLPK

LHTLHLQMNFI NQAQLSIFGTFRA

LRFVDLSDNRISGPST**LSEATPEEADDAEQEELLSADPHPAPLSTPASK**NFMDRCKNF

KFTMDLSRNNL VTIKPEMFVQLSR

LQCLSLSHNSI AQAVNGSQFLPLTN

LQVLDLSHNKL DLYHWKSFSELPQ

LQALDLSYNSQPF SMKGIGHQFSFVTHLSM

LQSLSLAHNDI HTRVSSHLNSNS

VRFLDFSGNGM GRMWDEGGLYLHFFQGLSG

LLKLDLSQNNL HILRPQNLDNLPKS

LKLLSLRDNYL SFFNWTSLSFLPN

LEVLDLAGNQL KALTQGTLPNGTL

LQKLDVSSNSI VSVVPAFFALAVE

LKEVNLSHNIL KTVDRSWFGPIVMQ

LTVLDVRSNPL HCACGAAFVDLLLEVQTKVPGLANGVKCGSPGQLQGRSIFAQDLRLCLDEVLSWDEFLVPR

>2Z7X_B

>TLR1

S

EFLVDRSKNGL IHVPKDLSQK

TTILNISQNYI SELWTSDILSLSK

LRILIISHNRI QYLDISVFKFNQE

LEYLDLSHNKL VKISCHPTVN

LKHLDLSFNAF DALPICKEFGNMSQ

LKFLGLSTTHL EKSSVLPIAHLN

ISKVLLVLGET YGEKEDPEGLQDFN

TESLHIVFPTN KEFHFILDVSVKT

VANLELSNIKC VLEDNKCSYFLSILAKLQTNPK

LSNLTLNNIETT WNSFIRILQLVWHTT

VWYFSISNVKL Q GQLDFRDFDYSGTS

LKALSIHQVVS 　 　DVFGFPQSYIYEIFSNMN

IKNFTVSGTRM VHMLCPSKISP

FLHLDFSNNLL TDTVFENCGHLTE

LETLILQMNQL KELSKIAEMTTQMKS

LQQLDISQNSV SYDEKKGDCSWTKS

LLSLNMSSNIL TDTIFRCLPPR

IKVLDLHSNKI KSIPKQVVKLEA

LQELNVASNQL KSVPDGIFDRLTS

LQKIWLHTNPW DCSCPRIDYLSRWLNKNSQKEQGSAKCSGSGKPVRSIICP

>2Z7X_A

>Human TLR2

LSCDRNGICKGSSGSLNSIPSGLTEA

VKSLDLSNNRI TYISNSDLQRCVN

LQALVLTSNGI NTIEEDSFSSLGS

LEHLDLSYNYL SNLSSSWFKPLSS

LTFLNLLGNPY KTLGETSLFSHLTK

LQILRVGNMDTF TKIQRKDFAGLTF

LEELEIDASDL QSYEPKSLKSIQN

VSHLILHMKQHI LLLEIFVDVTSS

VECLELRDTDL DTFHFSELSTGETNSL

IKKFTFRNVKI TDESLFQVMKLLNQISG

LLELEFDDCTL NGVGNFRASDNDRVIDPGK

VETLTIRRLHI PRFYLFYDLSTLYSLTER

VKRITVENSKV FLVPCLLSQHLKS

LEYLDLSENLM VEEYLKNSACEDAWPS

LQTLILRQNHL ASLEKTGETLLTLKN

LTNIDISKNSF HSMPETCQWPEK

MKYLNLSSTRI HSVTGCIPKT

LEILDVSNNNL NLFSLNLPQ

LKELYISRNKL MTLPDASLLPM

LLVLKISRNQL KSVPDGIFDRLTS

LQKIWLHTNPW DCSCPRIDYLSRWLNKNSQKEQGSAKCSGSGKPVRSIICP

>2Z81_A

>mouse TLR2

SLSCDA

SGVCDGRSRSF TSIPSGLTAA

MKSLDLSFNKI TYIGHGDLRACAN

LQVLILKSSRI NTIEGDAFYSLGS

LEHLDLSDNHL SSLSSSWFGPLSS

LKYLNLMGNPY QTLGVTSLFPNLTN

LQTLRIGNVETF SEIRRIDFAGLTS

LNELEIKALSL RNYQSQSLKSIRD

IHHLTLHLSES AFLLEIFADILSS

VRYLELRDTNL ARFQFSPLPVDEVSSP

MKKLAFRGSVL TDESFNELLKLLRYILE

LSEVEFDDCTL NGLGDFNPSESDVVSELGK

VETVTIRRLHI PQFYLFYDLSTVYSLLEK

VKRITVENSKV FLVPCSFSQHLKS

LEFLDLSENLM VEEYLKNSACKGAWPS

LQTLVLSQNHL RSMQKTGEILLTLKN

LTSLDISRNTF HPMPDSCQWPEK

MRFLNLSSTGI RVVKTCIPQT

LEVLDVSNNNL DSFSLFLPR

LQELYISRNKL KTLPDASLFPV

LLVMKISRNQL KSVPDGIFDRLTS

LQKIWLHTNPW DCSCPRIDYLSRWLNKNSQKEQGSAKCSGSGKPVRSIICP

>3A79_A

>TLR2

MLRALWLFWILVAITVLFSKRCSAQESLSCDA

SGVCDGRSRSF TSIPSGLTAA

MKSLDLSFNKI TYIGHGDLRACAN

LQVLILKSSRI NTIEGDAFYSLGS

LEHLDLSDNHL SSLSSSWFGPLSS

LKYLNLMGNPY QTLGVTSLFPNLTN

LQTLRIGNVETF SEIRRIDFAGLTS

LNELEIKALSL RNYQSQSLKSIRD

IHHLTLHLSES AFLLEIFADILSS

VRYLELRDTNL ARFQFSPLPVDEVSSP

MKKLAFRGSVL TDESFNELLKLLRYILE

LSEVEFDDCTL NGLGDFNPSESDVVSELGK

VETVTIRRLHI PQFYLFYDLSTVYSLLEK

VKRITVENSKV FLVPCSFSQHLKS

LEFLDLSENLM VEEYLKNSACKGAWPS

LQTLVLSQNHL RSMQKTGEILLTLKN

LTSLDISRNTF HPMPDSCQWPEK

MRFLNLSSTGI RVVKTCIPQT

LEVLDVSNNNL DSFSLFLPR

LQELYISRNKL KTLPDASLFPV

LLVMKIASNQL KSVPDGIFDRLTS

LQKIWLHTNPW DCSCPRIDYLSRWLNKNSQKEQGSAKCSGSGKPVRSIICPTLVPR

>3A79_B

>Mouse TLR6

MSQDRKPIVGSFHFVCALALIVGSMTPFSNELESMVDYSNRNLTHVPKDLPPR

TKALSLSQNSI SELRMPDISFLSE

LRVLRLSHNRI RSLDFHVFLFNQD

LEYLDVSHNRL QNISCCPMAS

LRHLDLSFNDF DVLPVCKEFGNLTK

LTFLGLSAAKF RQLDLLPVAH

LHLSCILLDLVSYHIKGGETESLQIPNTT

VLHLVFHPNSL FSVQVNMSVNALGHLQ

LSNIKLNDENC QRLMTFLSELTRGPT

LLNVTLQHIET TWKCSVKLFQFFWPRP

VEYLNIYNLTI TERIDREEFTYSETA

LKSLMIEHVKNQVFLFSKEALYSVFAEMN

IKMLSISDTPF IHMVCPPSPSS

FTFLNFTQNVF TDSVFQGCSTLKR

LQTLILQRNGL KNFFKVALMTKNMSS

LETLDVSLNSL NSHAYDRTCAWAES

ILVLNLSSNML TGSVFRCLPPK

VKVLDLHNNRI MSIPKDVTHLQA

LQELNVASNQL KSVPDGVFDRLTS

LQYIWLHDNPWDCTCPGIRYLSEWINKHSGVVRNS

AGSVAPDSAKCSGSGKPVRSIICPTLVPR

>2Z64_A

>Mouse TLR4

PCIEVVP

NITYQCMDQKL SKVPDDIPSS

TKNIDLSFNPL KILKSYSFSNFSE

LQWLDLSRCEI ETIEDKAWHGLHH

LSNLILTGNPI QSFSPGSFSGLTS

LENLVAVETKL ASLESFPIGQLIT

LKKLNVAHNFI HSCKLPAYFSNLTN

LVHVDLSYNYI QTITVNDLQFLRENPQV

NLSLDMSLNPI DFIQDQAFQGIK

LHELTLRGNFN SSNIMKTCLQNLAGLH

VHRLILGEFKDERNLEIFEPSIMEGLCDVT

IDEFRLTYTNDF SDDIVKFHCLAN

VSAMSLAGVSI KYLEDVPKHFK

WQSLSIIRCQL KQFPTLDLPF

LKSLTLTMNKGSI SFKKVALPS

LSYLDLSRNAL SFSGCCSYSDLGTNS

LRHLDLSFNGA IIMSANFMGLEE

LQHLDFQHSTL KRVTEFSAFLSLEK

LLYLDISYTN TKIDFDGIFLGLTS

LNTLKMAGNSF KDNTLSNVFANTTN

LTFLDLSKCQL EQISWGVFDTLHR

LQLLNMSHNNL LFLDSSHYNQLYS

LSTLDCSFNRI ETSKGILQHFPKS

LAFFNLTNNSVACICEHQKFLQWVKEQKQFLVNVEQMTCATPVEMNTSLVLDFNNSTC

>4G8A_A

>Human TLR4

RSPWDYKDDDDKLAAANSSIPESW

EPCVEVVP

NITYQCMELNF YKIPDNLPFS

TKNLDLSFNPL RHLGSYSFFSFPE

LQVLDLSRCEI QTIEDGAYQSLSH

LSTLILTGNPI QSLALGAFSGLSS

LQKLVAVETNL ASLENFPIGHLKT

LKELNVAHNLI QSFKLPEYFSNLTN

LEHLDLSSNKI QSIYCTDLRVLHQMPLL

NLSLDLSLNPM NFIQPGAFKEIR

LHKLTLRNNFDSL NVMKTCIQGLAGLE

VHRLVLGEFRNEGNLEKFDKSALEGLCNLT

IEEFRLAYLDYYL DGIIDLFNCLTN

VSSFSLVSVTI ERVKDFSYNFG

WQHLELVNCKF GQFPTLKLKS

LKRLTFTSNKG GNAFSEVDLPS

LEFLDLSRNGL SFKGCCSQSDFGTIS

LKYLDLSFNGV ITMSSNFLGLEQ

LEHLDFQHSNL KQMSEFSVFLSLRN

LIYLDISHTHT RVAFNGIFNGLSS

LEVLKMAGNSF QENFLPDIFTELRN

LTFLDLSQCQL EQLSPTAFNSLSS

LQVLNMSHNNF FSLDTFPYKCLNS

LQVLDYSLNHI MTSKKQELQHFPSS

LAFLNLTQNDF ACTCEHQSFLQWIKD

QRQLLVEVERMECATPSDKQGMPVLSLNITCQMTGHHHHHH

>3FXI_A

> Human TLR4

EPCVEVVP

NITYQCMELNF YKIPDNLPFS

TKNLDLSFNPL RHLGSYSFFSFPE

LQVLDLSRCEI QTIEDGAYQSLSH

LSTLILTGNPI QSLALGAFSGLSS

LQKLVAVETNL ASLENFPIGHLKT

LKELNVAHNLI QSFKLPEYFSNLTN

LEHLDLSSNKI QSIYCTDLRVLHQMPLL

NLSLDLSLNPM NFIQPGAFKEIR

LHKLTLRNNFDSLNVMKTCIQGLAGLE

VHRLVLGEFRNEGNLEKFDKSALEGLCNLT

IEEFRLAYLDYYLDDIIDLFNCLTN

VSSFSLVSVTI ERVKDFSYNFG

WQHLELVNCKF GQFPTLKLKS

LKRLTFTSNKG GNAFSEVDLPS

LEFLDLSRNGL SFKGCCSQSDFGTTS

LKYLDLSFNGV ITMSSNFLGLEQ

LEHLDFQHSNL KQMSEFSVFLSLRN

LIYLDISHTHT RVAFNGIFNGLSS

LEVLKMAGNSF QENFLPDIFTELRN

LTFLDLSQCQL EQLSPTAFNSLSS

LQVLNMSHNNF FSLDTFPYKCLNS

LQVLDYSLNHI MTSKKQELQHFPSS

LAFLNLTQNDF ACTCEHQSFLQWIKDQRQLLVEVER

MECATPSDKQGMPVLSLNITCQMNK

>3V47_A

>Zebra fish TLR5

ADPGTS

ECSVI

GYNAICINRGL HQVPELPAH

VNYVDLSLNSI AELNETSFSRLQD

LQFLKVEQQTPG LVIRNNTFRGLSS

LIILKLDYNQF LQLETGAFNGLAN

LEVLTLTQCNL DGAVLSGNFFKPLTS

LEMLVLRDNNI KKIQPASFFLNMRR

FHVLDLTFNKV KSICEEDLLNFQGKH

FTLLRLSSITLQDMNEYW LGWEKCGNPFKNTS

ITTLDLSGNGF KESMAKRFFDAIAGTK

IQSLILSNSYNMGSSFGHTNFKDPDNFTFKGLEASG

VKTCDLSKSKI FALLKSVFSHFTD

LEQLTLAQNEI NKIDDNAFWGLTH

LLKLNLSQNFL GSIDSRMFENLDK

LEVLDLSYNHI RALGDQSFLGLPN

LKELALDTNQL KSVPDGIFDRLTS

LQKIWLHTNPW DCSCPRIDYLSRWLNK

NSQKEQGSAKCSGSGKPVRSIICPTSASLVPR

>4QDH_A

>Mouse TLR9

ADPGGKACPSRCSCS

GTEIRCNSKGL TSVPTGIPSS

ATRLELESNKL QSLPHGVFDKLTQ

LTKLSLSRNNL VTIKPEMFVNLSR

LQCLSLSHNSI AQAVNGSQFLPLTN

LQVLDLSHNKL DLYHWKSFSELPQ

LQALDLSYNSQPFSMKGIGHNFSFVTHLSM

LQSLSLAHNDI HTRVSSHLNSNS

VRFLDFSGNGM GRMWDEGGLYLHFFQGLSG

LLKLDLSQNNL HILRPQNLDNLPKS

LKLLSLRDNYL SFFNWTSLSFLPN

LEVLDLAGNQL KALTNGTLPNGTL

LQKLDVSSNSI VSVVPAFFALAVE

LKEVNLSHNIL KTVDRSWFGPIVMN

LKELALDTNQL KSVPDGIFDRLTS

LQKIWLHTNPW DCSCPRIDYLSRWLNK

NSQKEQGSAKCSGSGKPVRSIICPTASLVPRGSWSHPQFEKGSHHHHHH

>4LXR_A

>Drosophila Toll receptor

SFGRDACSEMSIDGLCQCAPIMSEYEIICPANAENPTFRLTIQPKDYVQIMCNL

TDTTDYQQLPKKLRIGE

VDRVQMRRCML PGHTPIASILDYLGIVS

PTTLIFESDNL 　GMNITRQHLDRLHG

LKRFRFTTRRL 　THIPANLLTDMRN

LSHLELRANI 　EEMPSHLFDDLEN

LESIEFGSNKL 　RQMPRGIFGKMPK

LKQLNLWSNQL 　HNLTKHDFEGATS

VLGIDIHDNGI 　EQLPHDVFAHLTN

VTDINLSANLF 　 RSLPQGLFDHNKH

LNEVRLMNNRV 　PLATLPSRLFANQPE

LQILRLRAEL 　QSLPGDLFEHSTQ

ITNISLGDNLL 　KTLPATLLEHQVN

LLSLDLSNNRL 　THLPDSLFAHTTN

LTDLRLEDNLL 　TGISGDIFSNLGN

LVTLVMSRNRL 　RTIDSRAFVSTNG

LRHLHLDHNDIDLQQPLLDIMLQTQINSPFGYMHG

LLTLNLRNNSI 　IFVYNDWKNTMLQ

LRELDLSYNNI 　SSLGYEDLAFLSQN

RLHVNMTHNKI 　 RRIALPEDVHLGEGYNNN

LVHVDLNDNPL VCDCTILWFIQLV

RGVHKPQYSRQFKLRTDRLVCSQPNVLEGTPVRQIEPQTLICP

LDFSDDPRERKCPRGCNCHVRTYDK

ALVINCHSGNL THVPRLPNLHKNMQ

LMELHLENNTL LRLPSANTPGYES

VTSLHLAGNNL TSIDVDQLPTN

LTHLDISWNHL QMLNATVLGFLNRTMK

WRSVKLSGNPW MCDCTAKPLLLFTQ

DNFERIGDRNEMMCVNAEMPTRMVELSTNDICPAETGHHHHHH
